# A proteomic approach to understanding the pathogenesis of idiopathic macular hole formation

**DOI:** 10.1186/s12014-017-9172-y

**Published:** 2017-11-15

**Authors:** Pingbo Zhang, Min Zhu, Yuming Zhao, Jiang Qian, Craig Dufresne, Randi Turner, Richard D. Semba, Sharon D. Solomon

**Affiliations:** 1 Wilmer Eye Institute, Johns Hopkins University School of Medicine, Baltimore, MD USA; 2 National Institute on Aging, National Institutes of Health, Baltimore, MD USA; 3 Thermo Fisher Scientific, West Palm Beach, FL USA

**Keywords:** Eye, Idiopathic macular hole, Proteomics, Retina, Vitreous

## Abstract

Idiopathic macular holes (IMH) are full-thickness defects of retinal tissue that cause severe vision loss due to disruption of the anatomic fovea. Abnormal vitreous traction is involved in the formation of macular holes. Both glial cells and hyalocytes contribute to epiretinal membrane formation in IMH. In order to gain further insight into the pathophysiology of IMH, we conducted a discovery phase investigation of the vitreous proteome in four patients with macular holes and six controls using one-dimensional gel fractionation and liquid chromatography–tandem mass spectrometry analyses on an Orbitrap Elite mass spectrometer. Of a total of 5912 vitreous proteins, 32 proteins had increased and 39 proteins had decreased expression in IMH compared with controls, using a false discovery rate approach with p value < 0.001 and q value < 0.05. IMH was associated with increased expression of proteins in the complement pathway, α-2-macroglobulin, a major inducer of Müller glial cell migration, fibrinogen, and extracellular matrix proteins, and decreased expression of proteins involved in protein folding and actin filament binding. A proteomic approach revealed proteins and biological pathways that may be involved in the pathogenesis of IMH and could be targeted for future studies.

**Electronic supplementary material:**

The online version of this article (10.1186/s12014-017-9172-y) contains supplementary material, which is available to authorized users.

## Background

**Fig. 1:**
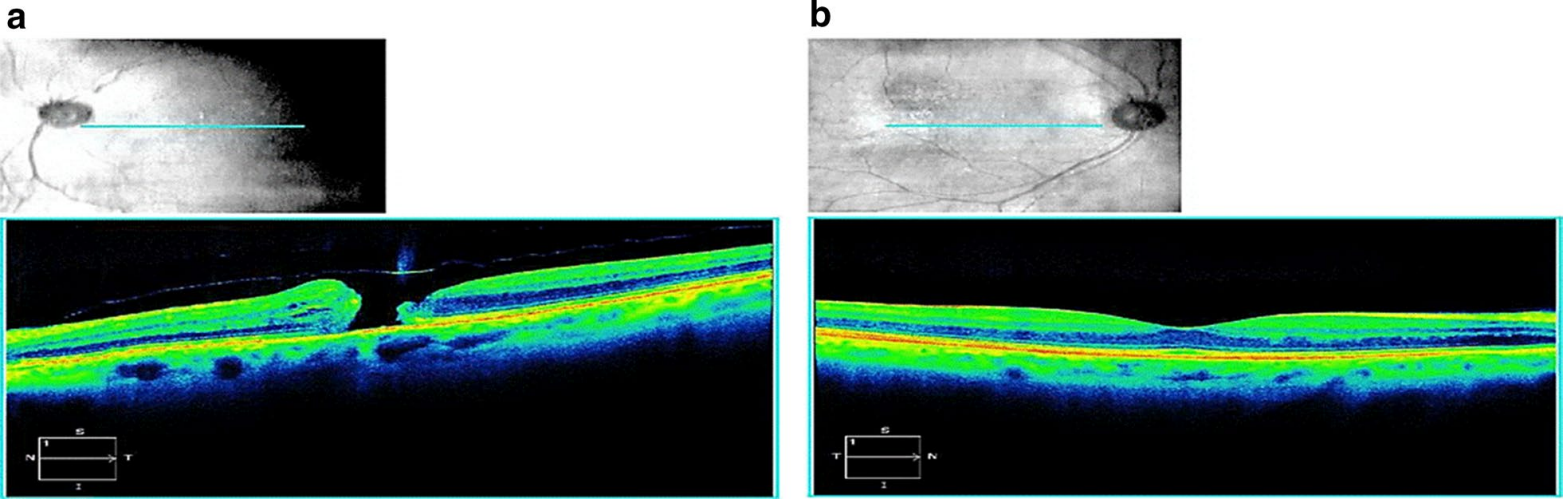
Zeiss spectral-domain image showing a full-thickness macular hole in the left eye (**a**) and a normal macula with good foveal contour in the right eye (**b**). Blue line in fundus image (above) denotes the plane of the cross-sectional image of the macula (below)

Idiopathic macular holes (IMH) are full-thickness defects of retinal tissue that are an important cause of severe, unilateral vision loss due to mechanical disruption of the anatomic fovea (Fig. 1). Most common in the sixth through eighth decades of life, macular holes are three times more likely to affect women than man [1, 2] and have up to a 14% risk of developing in the fellow eye [1]. An analysis of U.S. data recently showed that Asian-Americans are at higher risk of IMH [3].

Vitreoretinal traction is a central mechanism implicated in macular hole formation [4–6]. The role of epiretinal cell proliferation at the internal limiting membrane (ILM) remains controversial [7]. Activated glial cells, such as Müller cells and astrocytes, are considered a source of cells that migrate from the retina to the vitreous [8]. Conversely, it has been hypothesized that hyalocytes, macrophage-like cells in the vitreous, become activated and proliferate at the ILM and form epiretinal membranes [4]. Morphological criteria for identifying the originating cell types in epiretinal membranes has been difficult since cells undergo phenotypic transdifferentiation [9]. Recently, immunocytochemical and ultrastructural studies showed that epiretinal cell proliferation of both glial cells and hyalocytes occurs in the ILM at all stages of IMH [7]. Both cell migration and proliferation play a dominant role in the development of macular holes [7]. In patients with small full-thickness macular holes, single glial cells without proliferation or contraction were found on the ILM [10]. Larger clusters of cells which are positive for both glial and hyalocyte markers were also present on the ILM. These clusters may have greater potential for proliferation and exertion of tangential traction on the retina [10].

While treatment options exist for IMH, none are without significant associated morbidity. Surgical intervention with pars plana vitrectomy, possible internal limiting membrane peeling, and often intraocular gas tamponade may be associated with morbidity from prolonged face-down positioning, cataract progression, elevated eye pressure, retinal tear, and retinal detachment [11]. Ocriplasmin, a recombinant protease which has activity against fibronectin and laminin, is sometimes used for outpatient treatment of IMH, but treatment may be associated with decreased vision and abnormalities on electroretinogram testing [12].

Previous case series, including the Eye Disease Case–Control Study (EDCCS), have attempted to identify physiologic factors related to the risk of developing macular hole. The EDCSS observed a higher plasma fibrinogen concentrations in those affected with IMH compared to those without IMH [12]. Identification of the molecular abnormalities of the vitreous that predispose to IMH formation may lay the groundwork for the development of targeted therapies that could preclude macular hole formation at the earliest stages.

Proteomic analysis is a powerful tool for characterizing the diversity and abundance of proteins in both health and disease in tissues and cells and may provide new insights into the pathogenesis of IMH [13, 14]. We used a proteomic approach to identify proteins that are differentially expressed in IMH and to identify potential pathogenic mechanisms involved in IMH formation.

## Experimental procedures

We compared the vitreous proteome in four patients with IMH and six control patients with dislocated intraocular lens or epiretinal membrane. The mean age of cases and controls was 66.0 and 67.5 years, respectively. Adults in the study underwent standard of care pars plana vitrectomy for IMH, dislocated intraocular lens, or epiretinal membrane, and had no previous history of vitrectomy, Ocriplasmin injection, trauma, uveitis, or non-clearing vitreous hemorrhage in the eye undergoing surgery. Undiluted vitreous samples were collected from all patients.

### Preparation of vitreous samples

Vitreous samples were immediately snap frozen and stored at − 80 °C until processing. Vitreous was suspended in 1 ml of lysis buffer (10 mM HEPES, 42 mM KCl, 0.1 mM EDTA, 0.1 mM EGTA, 1.0 mM dithiothreitol [DTT], 1 × phosphatase inhibitor (Thermo Scientific, Waltham, MA, USA), 1 × protease inhibitor (Sigma Aldrich, St. Louis, MO, USA), and homogenized using a motorized handheld Eppendorf mortar/pestle. Samples were sonicated three times for 10 s and stored on ice to prevent overheating between sonications. Sodium dodecyl sulfate was added for a final concentration of 2% (w/v). Samples were incubated at room temperature (RT) for 10 min to extract the proteins. The samples were then spun for 45 min, 14,000×*g*, at RT. Protein concentration of the supernatant was determined using the Micro BCA Protein Assay kit (Thermo Scientific).

### Protein separation and fractionation

One-dimensional SDS-PAGE 4–12% gradient gels were used to separate proteins before double distilled H_2_O was added to the samples, resulting in 50 µg of protein in a 45 µl volume in a low retention Eppendorf tube. Forty-five µl 4 × lithium dodecyl sulfate SE + 100 mM DDT were added to tube, which was then vortexed, spun at 1000 rpm for 30 s at RT, and then placed on a 106 °C heat block for 10 min to denature the proteins. Tubes were again vortexed and spun twice at 1000 rpm for 30 s at RT. Fifty µg of sample were run on each lane of a 1D SDS gel, using NuPAGE 4–12% Bis–Tris gel 1.5 mm, 200 V. Coomassie blue (250 ml methanol, 50 ml acetic acid, 200 ml dH_2_O, 0.5 g Coomassie brilliant blue) was used to stain gels at RT for 30 min. Gels were then destained (50 ml methanol, 75 ml acetic acid, 875 ml d H_2_O) overnight. Each gel was cut into 12 bands. Gel bands were placed in separate vials and disrupted with forceps. Gel bands were then destained, dehydrated with acetonitrile, and dried down with a Savant SPD2010 Speedvac concentrator (Thermo Scientific) before samples were reduced with 10 mM DTT for 1 h at 55 °C and then alkylated with 55 mM iodoacetamide in 25 mM ammonium bicarbonate for 30 min at RT.

### Sample in-gel digestion and cleanup by C18 reverse column

The samples were then washed with ultrapure water, dehydrated with 100% acetonitrile, and dried by Speedvac concentrator before undergoing digestion with trypsin/LysC (V5073; ratio of protein to enzyme = 50:1, Promega, Madison, WI, USA) at 37 °C overnight with constant shaking. Supernatant with peptides was then collected into a new Eppendorf tube. The gel slices were incubated with 1% TFA v/v in 80% acetonitrile for extracting peptides twice and then combined for drying by Speedvac concentrator. Peptides were resuspended in solvent 0.1% v/v formic acid in water (Optima™ LC/MS, Thermo Scientific), desalted by the column (Spin-Tip, Thermo Scientific), spun, eluted with 0.1% v/v formic acid in 80% acetonitrile (Optima™ LC/MS, Thermo Scientific), and then dried by Speedvac concentrator. Finally, samples were resuspended in 0.1% formic acid in water and spiked with 5 fmol/µl calibration peptides (Peptide Retention Time Calibration Mixture, Thermo Scientific).

### Mass spectrometry analysis

Samples were run LC–MS/MS using a 1-h gradient of 2–30% acetonitrile (Fisher Scientific) with 0.1% formic acid (Sigma Aldrich) using an EASY-Spray source coupled with an Orbitrap Elite (Thermo Scientific) mass spectrometer. EASY-Spray source was run at 35 °C using a 25 cm × 75 μm integrated spray tip column. Peptides were trapped at 980 bar on a 2 cm × 75 μm trapping column. The trap was a 3 μm particle, and the column was 2 μm Acclaim PepMap C_18_. The Orbitrap instrument was operated in a positive ion mode, and the data-dependent product-ion mode was applied for all analyses. MS1 data were acquired with top 10 most intense spectra, at a resolution of 120,000, survey scans of *m*/*z* 400–2000 Th, and a target value of 1 × 10^6^. Peptide precursors with charge state 2–6 were sampled for MS2. Ion trap CID spectra were performed with an isolation width of 2.0 Th, 35% NCE, target value of 1 × 10^4^, and the max injection time was 100 ms. Dynamic exclusion was enabled with the following settings: repeat count = 1; repeat duration = 60 s; exclusion duration = 60 s; mass tolerance, ± 7 ppm. All individual samples were run with two technical replicates.

### Protein identification and quantification

MS raw files were batch processed using search engines of Mascot and X!Tandem and peak lists were searched against the UniProt Human Sequence database with 20,197 reviewed proteins. Mascot and X!Tandem were searched with a fragment ion mass tolerance of 0.10 Da and a parent ion tolerance of 50 PPM, allowing for 2 missed tryptic cleavages. Carbamidomethylation of cysteine was specified in Mascot and X!Tandem as a fixed modification. Deamidation of asparagine and glutamine, oxidation of methionine, acetylation of the n-terminus, carbamylation of lysine and phosphorylation of serine, threonine and tyrosine were specified as variable modifications.

Peptide identifications were accepted if they were established at > 95.0% probability by the Peptide Prophet algorithm with Scaffold Δ-mass correction. Protein identifications were accepted if they were established at > 99.0% probability and contained at least 2 identified peptides (minimum length of 7 amino acids). Protein Prophet algorithm was used to assign protein probabilities using a false discovery rate (FDR) of 0.2% for peptides and 1% for proteins against the target-decoy database. Proteins that contained similar peptides and could not be differentiated based on MS/MS analysis alone were grouped to satisfy the principles of parsimony. Proteins sharing significant peptide evidence were grouped into clusters. Scaffold 4.8.4 (Proteome Software Inc., Portland, OR, USA) was used for FDR filtering and to validate MS/MS-based peptide and protein identifications. The number of proteins and peptides that overlapped in the technical replicates were averaged to provide a mean for each sample. PANTHER analysis was used to classify protein function and to conduct pathway analyses. Relative quantification was performed using weighted spectral counting. Data log (2) transformed values were normalized based on total spectral counts per sample and compared using the tools described below.

### Bioinformatics analysis

An R package, DEGseq [15] was used to identify the differentially expressed proteins between samples from IMH and controls. p Values and q values were calculated using a FDR approach as described by Benjamini and Cohen [16] and Storey and Tibshirani [17]. FDR was used to control the proportion of false positives among potentially differentially expressed proteins, and the extended “q values” were defined as the minimum FDR that can be attained as p values have for type-I error control and calculated from the observed p values after estimating the proportion of differentially expressed proteins. An MA plot was used for visualization of proteomic data in significance analyses [18]. Using a p value < 0.001 and a cutoff of q value < 0.05, 71 differentially expressed proteins were identified. GO analyses were performed on these 71 proteins with DAVID (Database for Annotation, Visualization and Integrated Discovery). Protein–protein interaction networks were studied Ingenuity^®^ Pathway Analysis (QIAGEN Bioinformatics, Redwood City, CA, USA).

### Multiplexed selected reaction monitoring analysis

To validate for quantitative analysis by multiplexed selected reaction monitoring (SRM) assays, representative proteins with > 1.5-fold change were randomly selected and at least 2–3 of their tryptic peptides and optimization of transitions were selected following the guideline of Skyline (Seattle Proteome Center, Seattle, WA, USA.) (Additional file 1: Table S4). The heavy isotopically labeled tryptic fragment peptides were obtained from New England Peptide (Gardner, MA, USA) and prepared by Fmoc-based solid-phase peptide synthesis using per-^15^N, ^13^C-labeled (> 99% isotopic purity) Arg or Lys as the C-terminal residue attached to the resin. Peptides were purified by reversed phase chromatography (C18 stationary phase using water-acetonitrile gradients, ion-pairing agent ~ 0.1% TFA). The purity of the synthetic heavy peptides was ≥ 95% for each by the confirmation of analytical HPLC. Purified peptide solutions were prepared and the concentration of the solution was determined by amino acid analysis. Vitreous samples were carried out in-solution digestion in brief: 0.1 mg of proteins was aliquoted in 0.1% (w/v) RapiGest buffer containing 100 mM Tris–HCl, pH 8.0, and 100 mM dithiothreitol and incubated at 55 °C for 1 h for denaturation and reduction. The samples were then alkylated for 30 min at room temperature in dark with a final concentration of 50 mM iodoacetamide, digested by trypsin/LysC mix (Promega) at an enzyme-to-substrate ratio of 1:50 for 18 h at 37 °C. The digestion was terminated with 10% TFA to a final concentration of 1%, followed by centrifugation at 14,000×*g* for 15 min to remove RapiGest, and the sample was spiked with a heavy labeled peptide mixture at a concentration of 100 fmol/µl and cleaned up with a 96-well extraction plate vacuum manifold (Waters, Milford, MA, USA) according to manufacturer’s instruction.

SRM assays in triplicates were run on a 5500 QTrap (Sciex, Framingham, MA, USA) mass spectrometer equipped with an electrospray ionization source, a CBM-20A command module, LC-20ADXR pump, and a CTO-10Ac column oven heater (all Shimadzu, Kyoto, Japan). A sample volume of 10 µL was injected onto the column via a Shimadzu SIL-20AXR autosampler set to 4 °C at a flow rate of 0.2 ml/min, with an instrument run time of 18 min/sample including the 5 min column regeneration step. Chromatographic separations were conducted using an XBridge BEH C18 Column, 130 Å, 3.5 µm, 2.1 mm × 100 mm (Waters), with a linear gradient starting from 5% phase B increasing to 36% phase B within 10 min with the column oven set at 37 °C. Mobile phases consisted of water containing 0.1% formic acid (phase A) and 98% acetonitrile containing 0.1% formic acid (phase B). The mass spectrometry analysis was performed in positive ion mode and performed at unit resolution in both Q1 and Q3 quadrupoles. All sample data were collected using Analyst 1.5 software and processed using MultiQuant software version 3.02 (Sciex) with the parameters: Gaussian smooth width = 0, minimum peak width of points = 3, noise percentage = 40.0%, baseline subtraction window = 2.00 min, and peak splitting points = 2. All raw peak area ratios were calculated by contrasting against the heavy standard peptides and normalized by albumin. ANOVA post hoc tests were used for statistical analysis and validation of protein changes between cases (n = 12) and controls (n = 18).

## Results

In vitreous, the primary searching generated 6054 clusters and 6393 proteins. There were 5912 non-redundant proteins and 326,434 spectra (Additional file 2: Table S1), after removing immunoglobulins (Ig fragments) and redundant proteins. The change in an MA plot of IMH versus control proteins is shown in Fig. 1, with red points identified as differentially expressed. There were 32 proteins with increased expression and 39 proteins with decreased expression in IMH compared with controls. The 1.5-fold change in expression between IMH and controls are shown in Additional file 3: Table S2 and Additional file 4: Table 3.

**Fig. 2:**
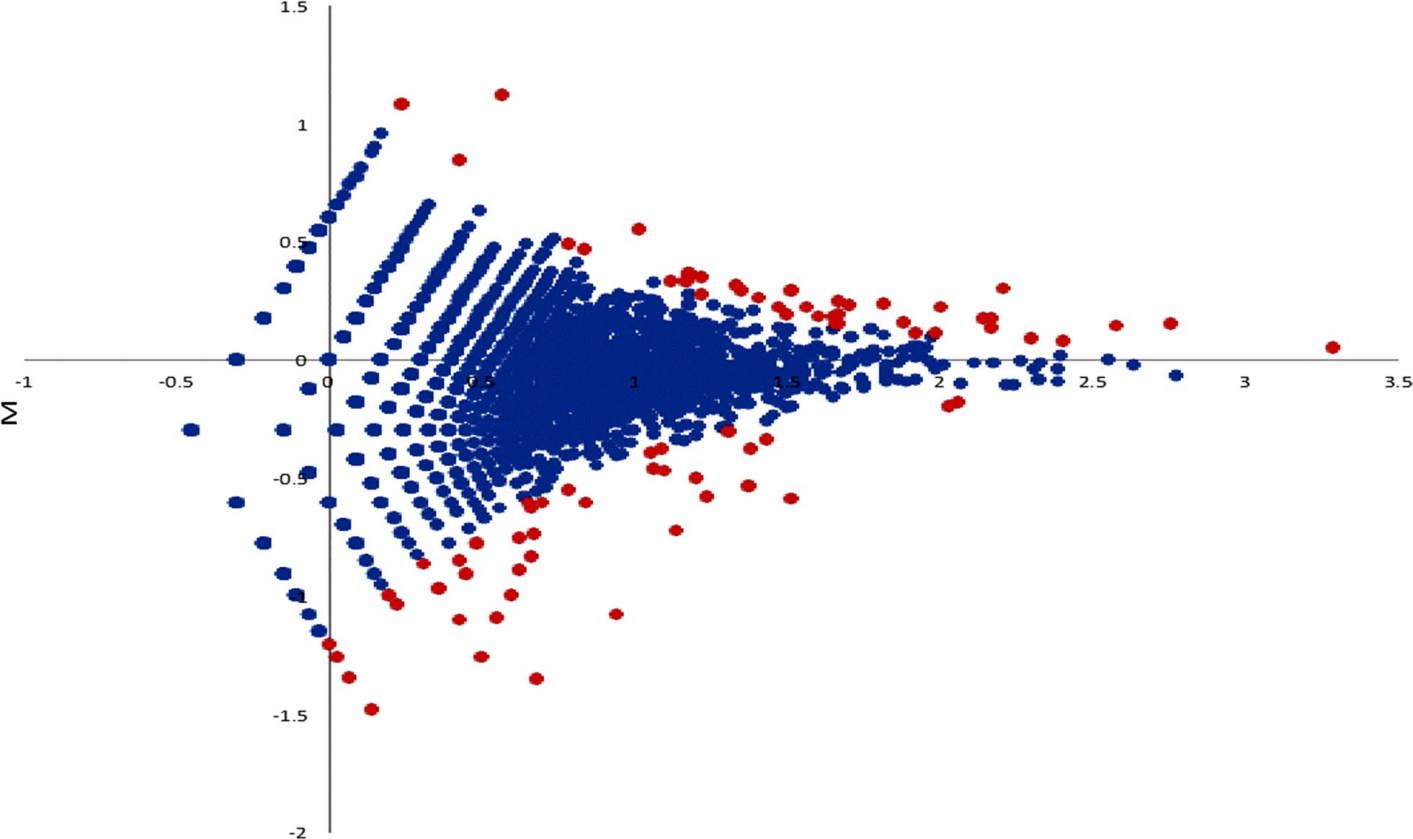
Significance analysis of vitreous proteins in IMH versus control. MA plot of human vitreous proteins [15, 18]. X-axis shows the average protein expression level, and Y-axis shows the fold change between IMH and control (in log_2_ scale). Each point represents a protein detected in the MS assay. The differentially expressed proteins were highlighted in red

Proteins with increased expression in the vitreous of IMH compared with controls (Table 1) were involved in the complement pathway (complement factor H, complement factor B, complement C3, complement C4-A), extracellular matrix (ECM) (brevican core protein, spondin-1, retinol-binding protein 3, versican core protein, collagen α-1(II) chain, target of Nesh-SH3), hyaluronan binding (inter-α-trypsin inhibitor heavy chain H1, inter-α-trypsin inhibitor heavy chain H2, inter-α-trypsin inhibitor heavy chain H4), and fibrinogen (Fig. 2).

**Table 1:** Thirty-two proteins with increased expression in the vitreous of idiopathic macular holes compared with controls

Protein name	Gene	neXtProt ID	Function
Melanotransferrin	MELTF	P08582	Iron ion binding
Brevican core protein	PGCB	Q96GW7	Hyaluronan binding; ECM component; involved in cell adhesion
Neurexin-2	NRXN2	Q9P2S2	Neuronal cell surface protein involved in cell recognition and cell adhesion
Ubiquitin-associated protein 2	UBAP2	Q5T6F2	Cadherin binding involved in cell–cell adhesion
Spondin-1	SPON1	Q9HCB6	Cell adhesion protein; found in ECM
Apolipoprotein B-100	APOB	P04114	Phospholipid/cholesterol transporter activity
Metallothionein-1A	MT1A	P04731	Zinc ion binding; negative regulation of growth
Retinol-binding protein 3	RBP3	P10745	Serine-type peptidase activity; found in ECM between retina and RPE
Fibrinogen gamma chain	FGG	P02679	Together with fibrinogen α and β, polymerizes to form an insoluble fibrin matrix
Complement factor H	CFH	P08603	Acts as cofactor in the inactivation of the alternative complement pathway
Collagen α-2(IX)chain	COL92A	Q14055	Structural component of the vitreous
Versican core protein	VCAN	P13611	Intracellular signaling and connecting cells with ECM; binds hyaluronan
Amyloid-like protein 2	APLP2	Q06481	Serine-type endopeptidase inhibitor activity
Inter-α-trypsin inhibitor heavy chain H2	ITIH2	P19823	Involved as binding protein between hyaluronan and other matrix proteins
Inter-α-trypsin inhibitor heavy chain H4	ITIH4	Q14624	Type II acute phase response protein; serine-type endopeptidase inhibitor activity
Collagen α-1(II) chain	COL2A1	P02458	Extracellular matrix structural constituent conferring tensile strength
Target of Nesh-SH3	ABI3BP	Q7Z7G0	Collagen binding; ECM organization; positive regulator of cell-substrate adhesion
Complement factor B	CFB	P00751	Role in alternative complement pathway; cleaved by factor D into Ba and Bb fragments
Apolipoprotein A-IV	APOA4	P06727	Component of chylomicrons, HDL; cholesterol and lipid transport
Inter-α-trypsin inhibitor heavy chain H1	ITIH	P19827	Carrier of hyaluronan or as binding protein between hyaluronan and other matrix proteins
Haptoglobin	HP	P00738	Antioxidant activity
α-2-Macroglobulin	A2 M	P01023	Inhibits all four classes of proteinases
Fibrinogen α chain	FGA	P02671	Polymerizes with fibrinogen β and γ to form an insoluble fibrin matrix
Calsyntenin-1	CLSTN1	O94985	Interacts with amyloid precursor protein, kinesin-1
Apolipoprotein A-I	APOA1	P02647	Cholesterol transport
Serotransferrin	TF	P02787	Iron binding transport protein
Complement C3	C3	P01024	Central role in activation of the complement system
Ceruloplasmin	CP	P00450	Copper-binding glycoprotein; ferroxidase activity in oxidizing Fe^2+^ to Fe^3+^
Gelsolin	GSN	P06396	Role in actin filament capping and polymerization
Pigment epithelium-derived factor	SERPINF1	P36955	Inhibitor of angiogenesis; neurotrophic
Complement C4-A	C4A	P0C0L4	Nonenzymatic component of C3 and C5 convertases; essential for propagation of classical complement pathway
Serum albumin	ALB	P02768	Main plasma protein; binds water, Ca^2+^, Na^+^, K^+^, zinc, fatty acids, etc.

Differentially expressed proteins with the normalized log2 fold change at *p* < 0.001 and q value < 0.05

Proteins with decreased expression in the vitreous of IMH compared with controls (Table 2) included those involved in protein folding (T-complex protein 1 subunit ζ, heat shock protein β-1, heat shock 70 kDa protein 1A, UDP-glucose:glycoprotein), actin filament binding (filamin-A, vinculin), cell adhesion (transforming growth factor-β-induced protein ig-h3, transgelin-2, myocilin), crystallin proteins (α-crystallin B chain, α-crystallin A chain, β-crystallin A3, and β-crystallin B2), and glycolytic enzymes (phosphoglycerate kinase 1, α-enolase).

**Table 2:** Thirty-nine proteins with decreased expression in the vitreous of idiopathic macular holes compared with controls

Protein name	Gene	neXtProt ID	Function
Stromelysin-1	MMP3	P08254	Degrades fibronectin; involved in breakdown and remodeling of ECM proteins
Transforming growth factor-β-induced protein ig-h3	TGFBI	Q15582	Plays a role in cell adhesion; involved in cell–collagen interactions
T-complex protein 1 subunit zeta	CCT6A	P40227	Molecular chaperone; assists in the folding of proteins, including actin and tubulin
RNA-binding protein 43	RBM43	Q6ZSC3	Nucleotide and RNA binding
Vimentin	VIM	P08670	Class-III intermediate filament; normal component of vitreous
Pro-neuregulin-1, membrane-bound isoform	NRG1	Q02297	Tyrosine kinase activator activity; ligand for integrins
α-Crystallin B chain	CRYAB	P02511	Normal component of vitreous, lens, cornea; molecular chaperone
α-Crystallin A chain	CRYAA	P02489	Normal component of vitreous, lens, cornea; molecular chaperone
Mixed lineage kinase domain-like protein	MLKL	Q8NB16	Pseudokinase with role in TNF-induced necroptosis, a programmed cell death process
Retinal dehydrogenase 1	ALDH1A1	P00352	Converts retinaldehyde to retinoic acid; binds retinal
Heat shock protein β-1	HSPB1	P04792	Involved in stress resistance and actin organization
Lipocalin-1	LCN1	P31025	Cysteine-type endopeptidase inhibitor activity
Phosphoglycerate kinase 1	PGK1	P00558	Glycolytic enzyme
CLOCK-interacting pacemaker	CIPC	Q9C0C6	Transcriptional repressor
Glucoside xylosyltransferase 2	GXYLT2	A0PJZ3	Glycosyltransferase which elongates the O-linked glucose attached to EGF-like repeats in the extracellular domain of Notch proteins by catalyzing the addition of xylose
Carbonic anhydrase 1	CA1	P00915	Reversible hydration of carbon dioxide
Leucine-rich repeat and IQ domain-containing protein 3	LRRIQ3	A6PVS8	Protein binding
Transgelin-2	TAGLN2	P37802	Regulates cell migration; cadherin binding involved in cell–cell adhesion
β-Crystallin A3	CRYBA1	P05813	Normal component of vitreous, lens, cornea
Decorin	DCN	P07585	Extracellular matrix binding; glycosaminoglycan binding; may affect rate of fibrils formation
Tetraspanin-16	TSPAN16	Q9UKR8	Cell surface receptor signaling pathway
Taste receptor type 2 member 9	TAS2R9	Q9NYW1	Taste receptor activity
SLIT and NTRK-like protein 3	SLITRK3	O94933	Suppresses neurite outgrowth; axonogenesis
β-Crystallin B2	CRYBB2	P43320	Normal component of vitreous, lens, cornea
DNA-directed RNA polymerase III subunit RPC2	POLR3B	Q9NW08	Contributes to RNA polymerase III activity
55 kDa erythrocyte membrane protein	MPP1	Q00013	Regulates neutrophil polarity
α-Enolase	ENO1	P06733	Plays a role in glycolysis; role in fibrinolytic system as receptor and activator of plasminogen; cadherin binding involved in cell–cell adhesion
Heat shock 70 kDa protein 1A	HSPA1A	P0DMV8	Stabilizes preexistent proteins against aggregation in cooperation with other chaperones
Apolipoprotein D	APOD	P05090	Lipid transport
Filamin-A	FLNA	P21333	Involved in actin filament binding; promotes orthogonal branching of actin filaments; links actin filaments to membrane glycoproteins
Myocilin	MYOC	Q99972	Secreted glycoprotein that regulates activation of different signaling pathways involved in cell adhesion, cell–matrix adhesion, cytoskeleton organization, and cell migration; binds with fibronectin
UDP-glucose:glycoprotein glucosyltransferase 2	UGGT2	Q9NYU1	Recognizes glycoproteins with minor folding defects; provide quality control for protein folding in the endoplasmic reticulum
Uncharacterized protein C1orf167	C1orf167	Q5SNV9	Unknown
Vinculin	VCL	P18206	Actin filament binding protein involved in cell matrix adhesion and cell–cell adhesion; regulates cell-surface E-cadherin expression
Dynein heavy chain 1, axonemal	DNAH1	Q9P2D7	Cilium movement involved in cell motility
β-Actin-like protein 2	ACTBL2	Q562R1	Major constituent of the contractile apparatus
Tectonic-1	TCTN1	Q2MV58	Cilium assembly
Hemoglobin subunit β	HBB	P68871	Oxygen transport; contributes to haptoglobin binding
Potassium voltage-gated channel subfamily G member 1	KCNG1	Q9IUX4	Potassium channel activity

Differentially expressed proteins with the normalized log2 fold change at *p* < 0.001 and q value < 0.05

We used SRM to validate differentially expressed proteins. Thirteen proteins with 1.5-fold changes were assayed and validated with significant differential expressions between cases (n = 12) and controls (n = 18) (Additional file 5: Figures S1–S25).

**Fig. 3:**
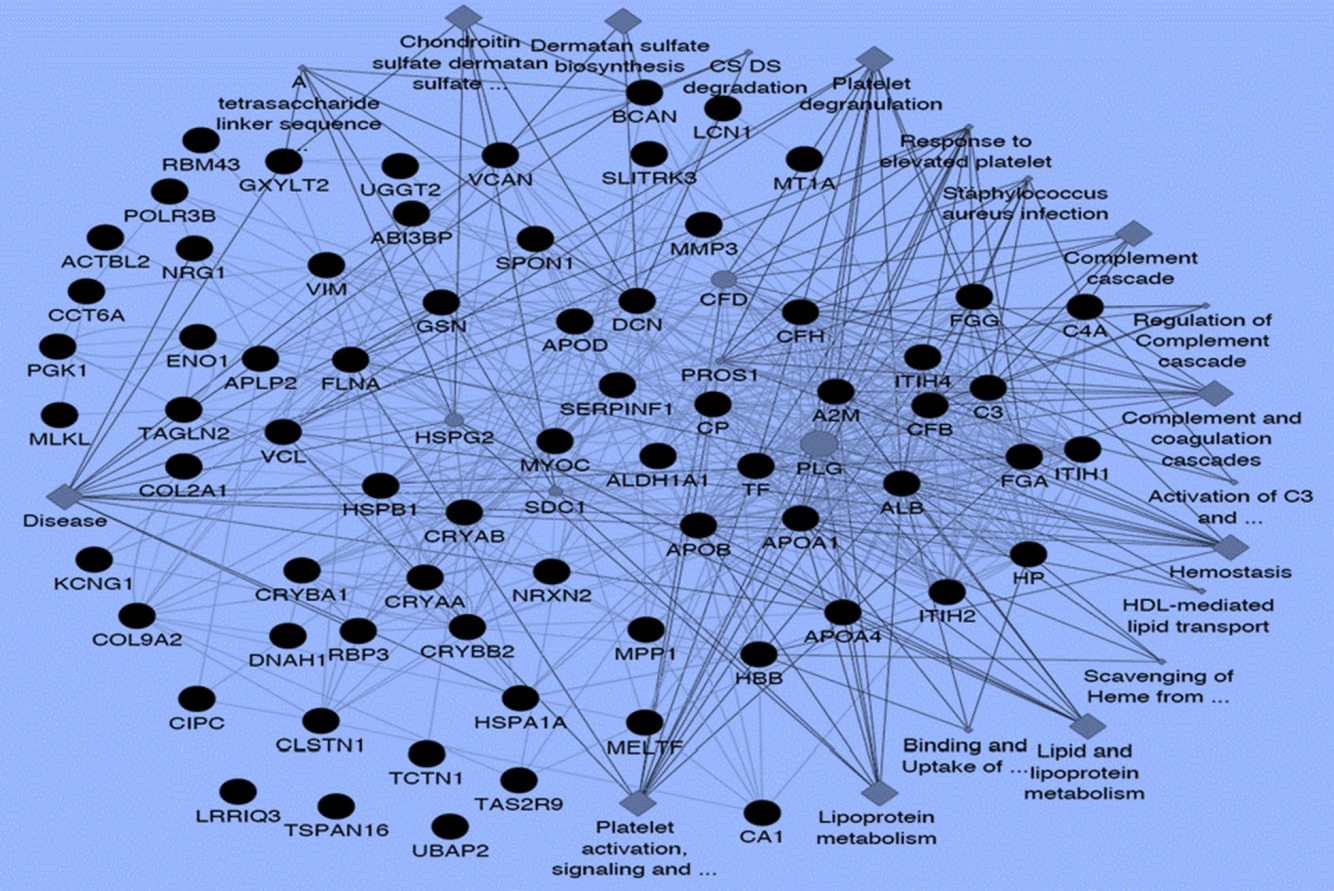
Protein–protein interaction network. The network weighting method is based on GO biological process. Black nodes represent genes associated with the biological process, diamond represents the biological process and the edge represents the type and strength of gene interaction. Biological processes include: binding and uptake of ligands by scavenger receptors, scavenging of heme from plasma, platelet activation, signaling and aggregation, activation of C3 and C5, chondroitin sulfate dermatan sulfate metabolism, and A tetrasaccharide linker sequence is required for GAG synthesis

Protein–protein interactions among vitreous proteins that are differentially expressed between IMH and controls are shown in Fig. 3. GO processes that were identified include metabolism of chondroitin sulfate and dermatan sulfate (versican, brevican), platelet degranulation (fibrinogen γ chain, gelsolin, metallothionein-1A), complement cascade (complement factor H, complement factor B, complement C3, complement C4-A), scavenging of heme from plasma (haptoglobin, hemoglobin beta), lipoprotein metabolism (apolipoprotein A-I, apolipoprotein A-IV, apolipoprotein B-100, apolipoprotein D).

## Discussion

The present proteomic investigation of the vitreous identifies proteins, pathways, and biological processes that could be involved in the pathogenesis of IMH, including the complement pathway, induced migration of Müller glial cells, fibrinogen, ECM proteins, protein folding, and actin filament binding, as presented in greater detail below.

The complement system of innate immunity is involved in the recognition of diseased or damaged host cells, regulation of cellular immune responses, and interaction with the coagulation cascade [19]. Complement—considered a “master of sensing”—can discriminate between foreign, altered, and healthy surfaces, but excessive activation or insufficient control of the complement system can lead to an imbalance that exacerbates the pathology of disease. Two proteins of the alternative complement pathway, complement factors H and B, were upregulated in the vitreous from eyes with IMH compared with controls. In addition, complement C3, which plays a central role in the activation of the complement system, was also upregulated. The increased expression of complement C4-A in IMH also suggests involvement of the classical complement pathway.

The migration of Müller glial cells from the retina to the vitreal surface has been implicated in the pathogenesis of IMH formation [8]. α-2-macroglobulin (α_2_M), a major antiproteinase in plasma and fluids, was increased in the vitreous of patients with IMH. α_2_M inhibits a broad spectrum of proteases [20]. α_2_M consists of two noncovalently bound dimers of disulfide-linked identical subunits. Near the middle of the polypeptide chain is a unique sequence of amino acids, known as the “bait” region, which is susceptible to cleavage by proteinases. Cleavage by proteinases introduces a conformational change and activation of α_2_M [20]. Activated α_2_M can specifically bind to low-density lipoprotein receptor-related protein 1 (LRP1), which induces Müller glial cell migration [21]. Increased expression of α_2_M is a possible mechanism by which Müller glial cell may acquire the ability to migrate to the vitreous in the formation of IMH.

A multicenter epidemiological study from the EDCCS involving 198 cases of IMH and 1023 controls showed that elevated circulating fibrinogen concentrations were a strong independent risk factor for IMH [13]. The underlying basis for this unexpected epidemiological association was unclear. It was speculated that elevated fibrinogen could possibly compromise blood flow in the macular region or act upon the vitreous in an unknown way that increases the susceptibility of the macula to vitreous traction [13]. In the present study, there was increased expression of fibrinogen α and γ chains in the vitreous of patients with IMH compared with controls. These observations appear to corroborate and extend the findings from the EDCCS. In an animal model, fibrinogen induced the formation of vitreous membranes when injected into the vitreous [22]. Fibrinogen may be transformed into a long fibrin polymer that forms a matrix that allows surrounding cells to proliferate in the vitreous [22].

Five protein components of the extracellular matrix (brevican core protein, spondin-1, retinol-binding protein 3, versican core protein, collagen α-1(II) chain, target of Nesh-SH3) and three proteins involved in binding with hyaluronan, a major constituent of the extracellular matrix (inter-α-trypsin inhibitor heavy chain H1, inter-α-trypsin inhibitor heavy chain H2, inter-α-trypsin inhibitor heavy chain H4), were upregulated in IMH compared with controls. The ECM undergoes extensive remodeling during cell proliferation, differentiation, and migration, and such changes can determine the course of disease pathogenesis [23]. Versican and fibrinogen bind with hyaluronan in the ECM [23]. Members of the inter-α-trypsin inhibitor proteoglycan family covalently bind to hyaluronan and stabilize the ECM [24]. These findings suggest that the ECM undergoes active remodeling during the formation of IMH.

Six molecular chaperone proteins involved in the proper folding of proteins were downregulated in IMH: T-complex protein 1 subunit ζ, heat shock protein β-1, heat shock 70 kDa protein 1A, UDP-glucose:glycoprotein, α-crystalline A chain, and α-crystalline B chain. T-complex protein 1 subunit ζ folds various proteins, including actin [25]. Heat shock protein β-1 plays a role in proper assembly of actin filaments [26]. Heat shock 70 kDa protein 1A prevents the aggregation of misfolded proteins and can convert misfolded proteins into active conformation [27]. UDP-glucose: glycoprotein recognizes glycoproteins that have minor folding defects [28]. α-crystalline A chain and α-crystalline B chain have chaperone-like activity and may prevent aggregation of various proteins under conditions of stress. The downregulation of these molecular chaperone proteins suggests that surveillance for misfolded proteins may be compromised in IMH. Two actin filament binding proteins, filamin-A and vinculin, had lower expression in IMH compared with controls. Vinculin plays a role in binding and rearranging the actin cytoskeleton [29]. Filamin-A plays a role in cross-linking and stabilizing the F-actin cytoskeleton [30].

## Conclusions

This study showed that the complement pathway, α_2_M, an inducer of Müller glial cell migration, extracellular matrix remodeling, fibrinogen, molecular chaperones involved in protein folding, and actin filament binding proteins may play a role in the pathogenesis of IMH.

## Additional files

**Additional file 1: Table** **4.** SRM assay for protein changes related to the pathogenesis of human idiopathic macular hole.

**Additional file 2: Table** **1.** Proteins identified in the vitreous.

**Additional file 3: Table** **2.** Differentially expressed proteins in the vitreous between idiopathic macular hole and controls.

**Additional file 4: Table** **3.** The 1.5-fold change in protein expression between IMH and controls.

**Additional file 5. Figures S1–S25.** SRM assay to validate differentially expressed proteins.
