# Supplementary material for: A proteomic approach to understanding the pathogenesis of idiopathic macular hole formation
Source: Clin Proteomics. 2017 Nov 15;14:37. doi: 10.1186/s12014-017-9172-y (PMC5688700; doi:10.1186/s12014-017-9172-y)
Supplement: Supplementary file 5 — Additional file 5. Figures S1–S25. SRM assay to validate differentially expressed proteins. [file 12014_2017_9172_MOESM5_ESM.pptx]

## Slide 1
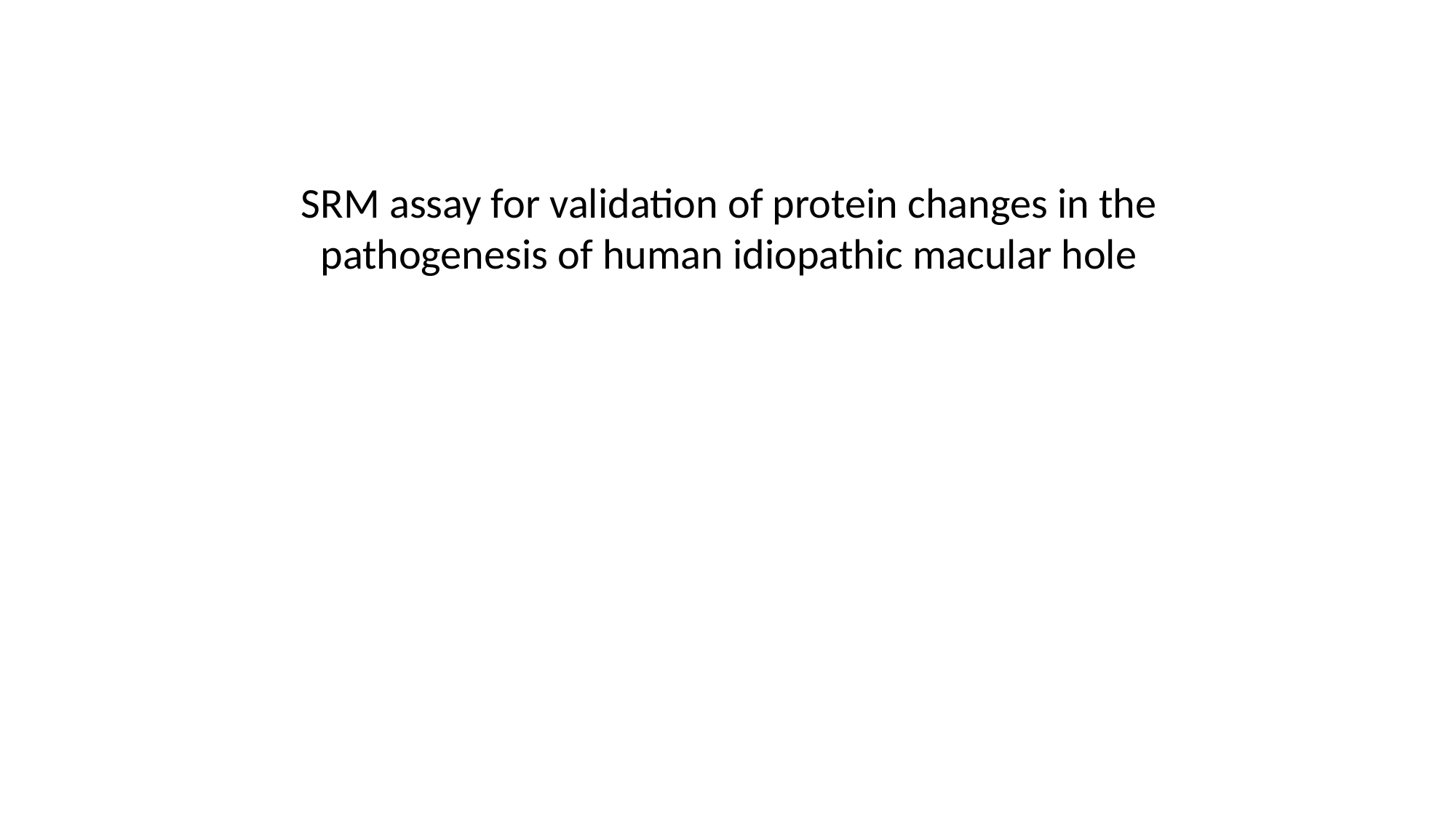

SRM assay for validation of protein changes in the pathogenesis of human idiopathic macular hole

## Slide 2
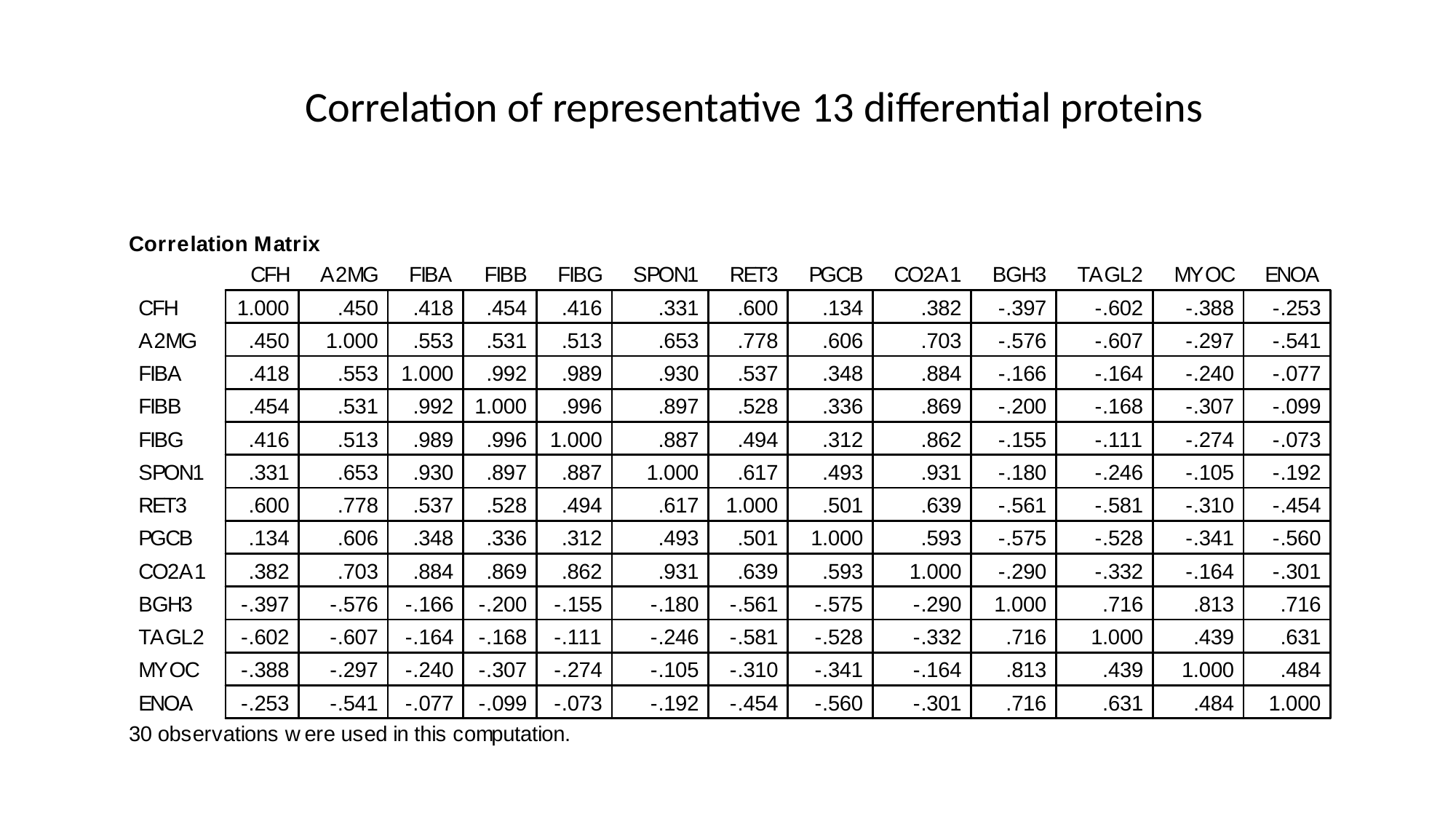

Correlation of representative 13 differential proteins

## Slide 3
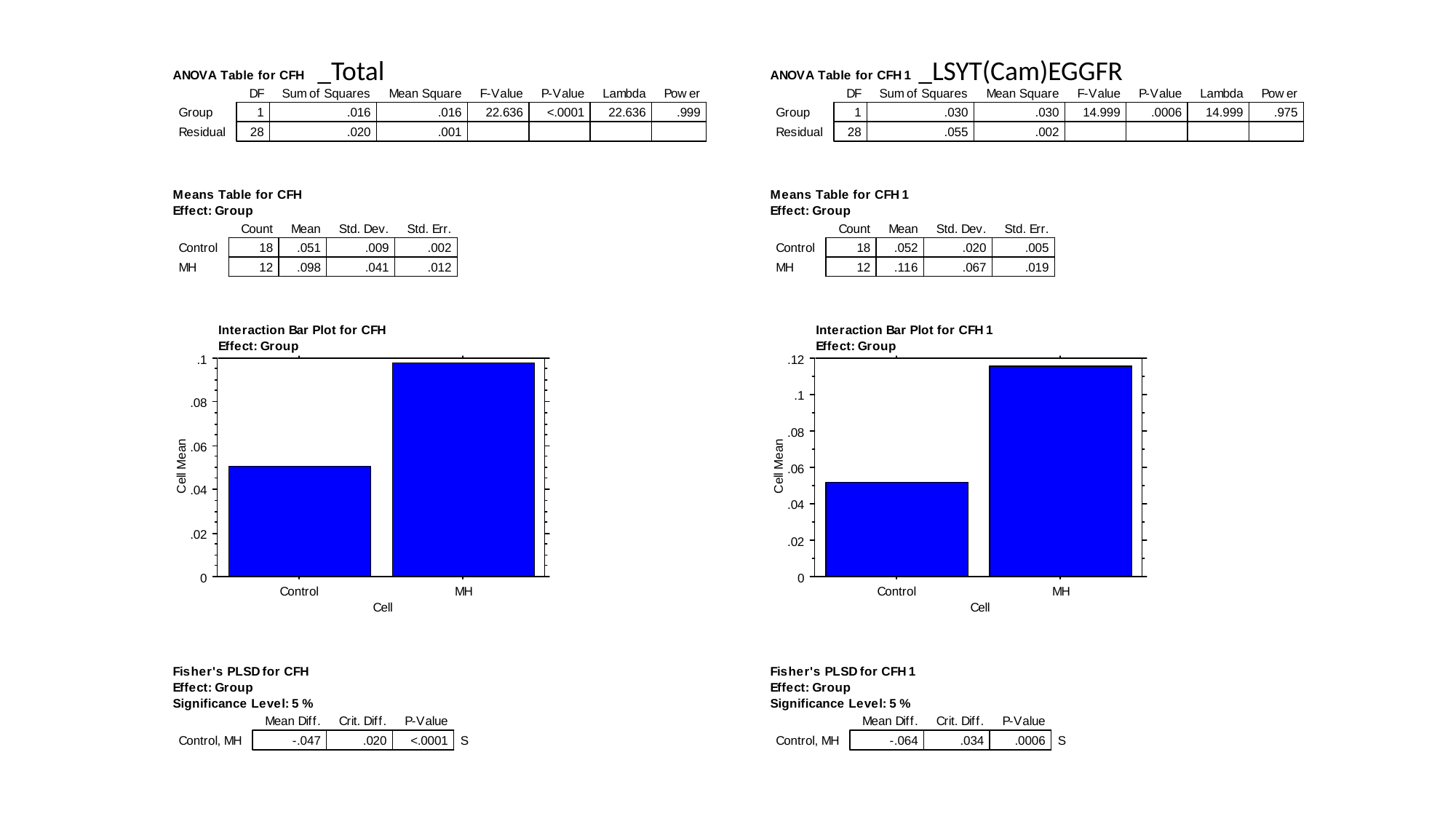

_Total
_LSYT(Cam)EGGFR

## Slide 4
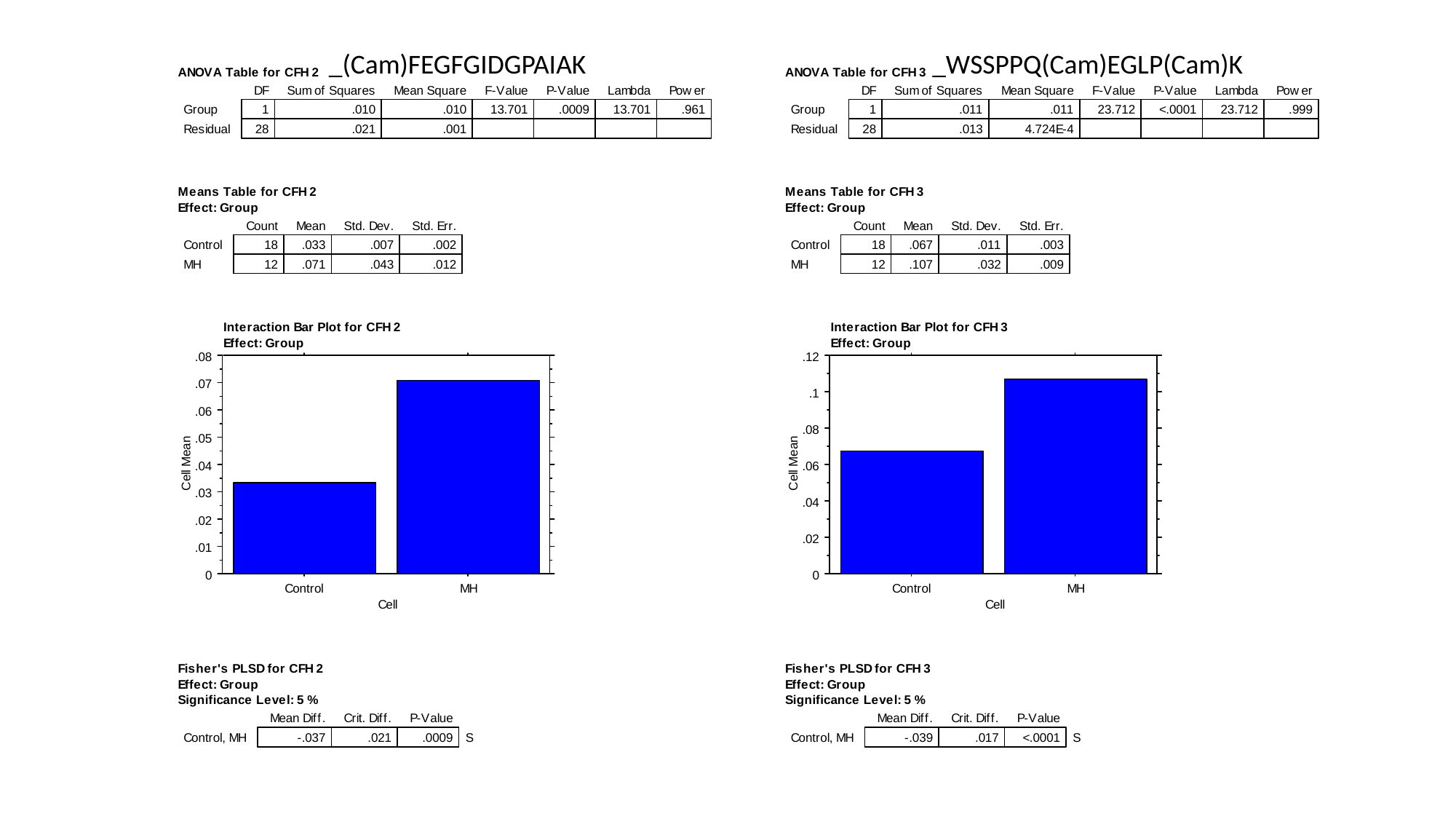

_(Cam)FEGFGIDGPAIAK
_WSSPPQ(Cam)EGLP(Cam)K

## Slide 5
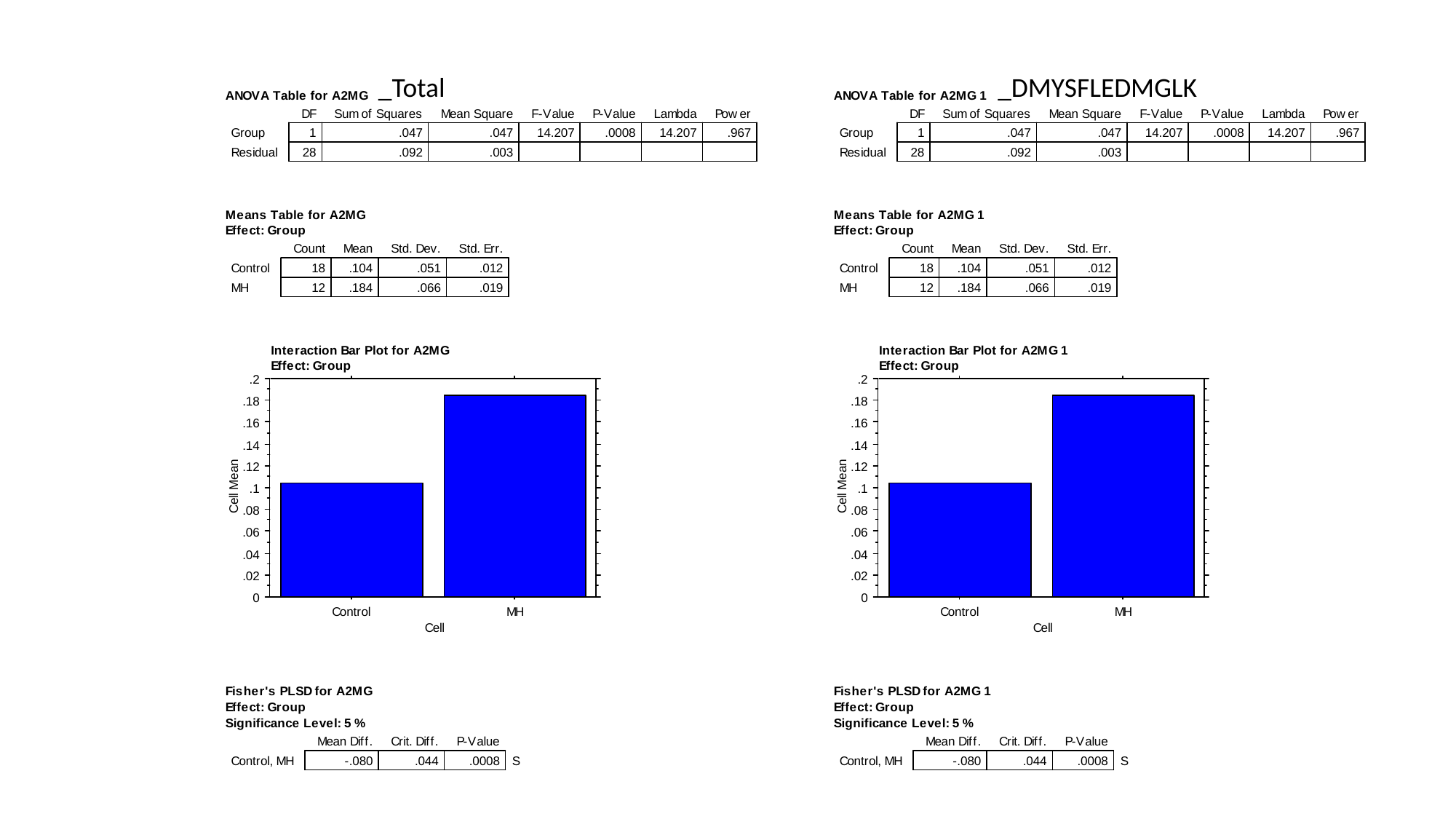

_Total
_DMYSFLEDMGLK

## Slide 6
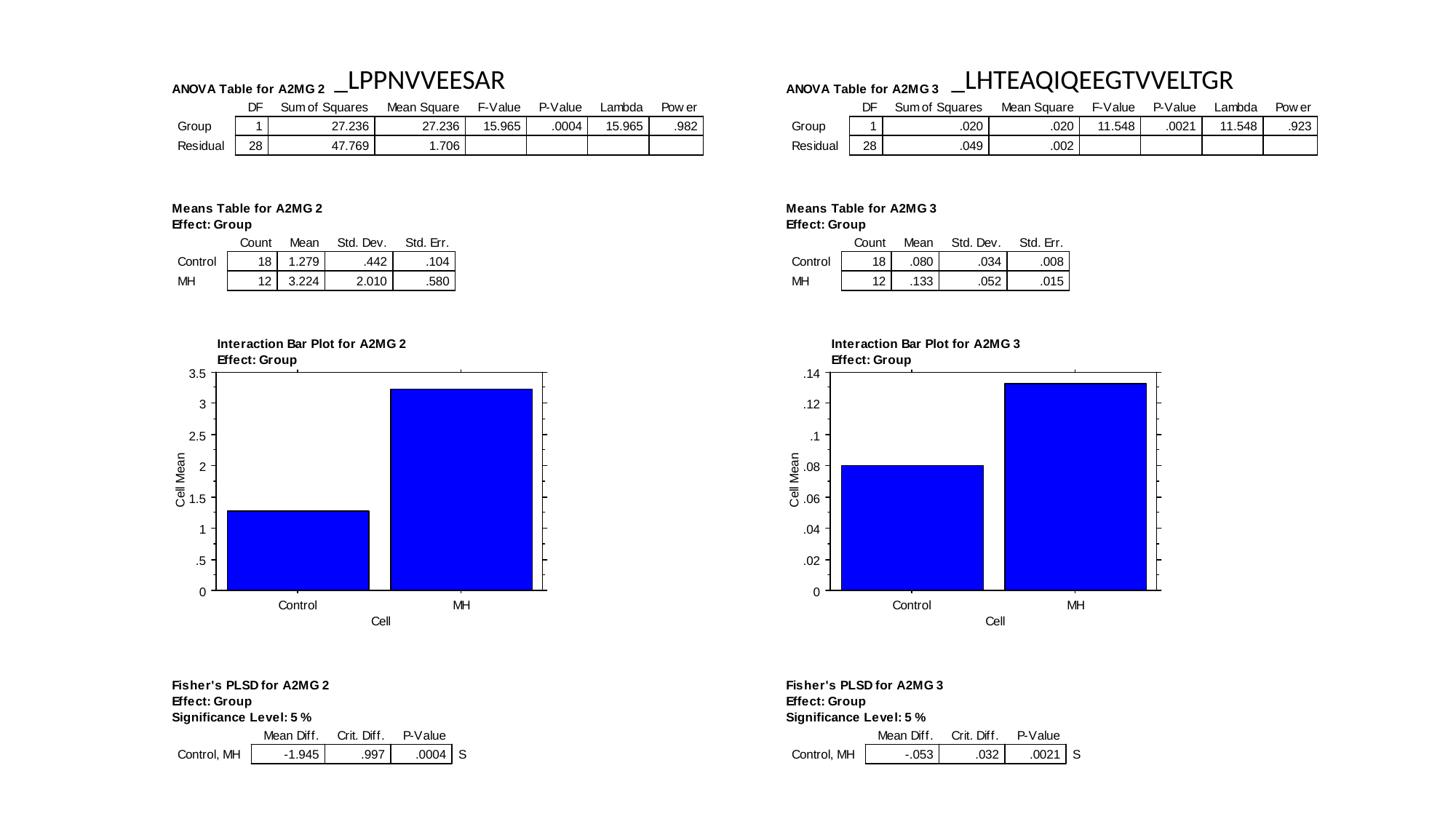

_LPPNVVEESAR
_LHTEAQIQEEGTVVELTGR

## Slide 7
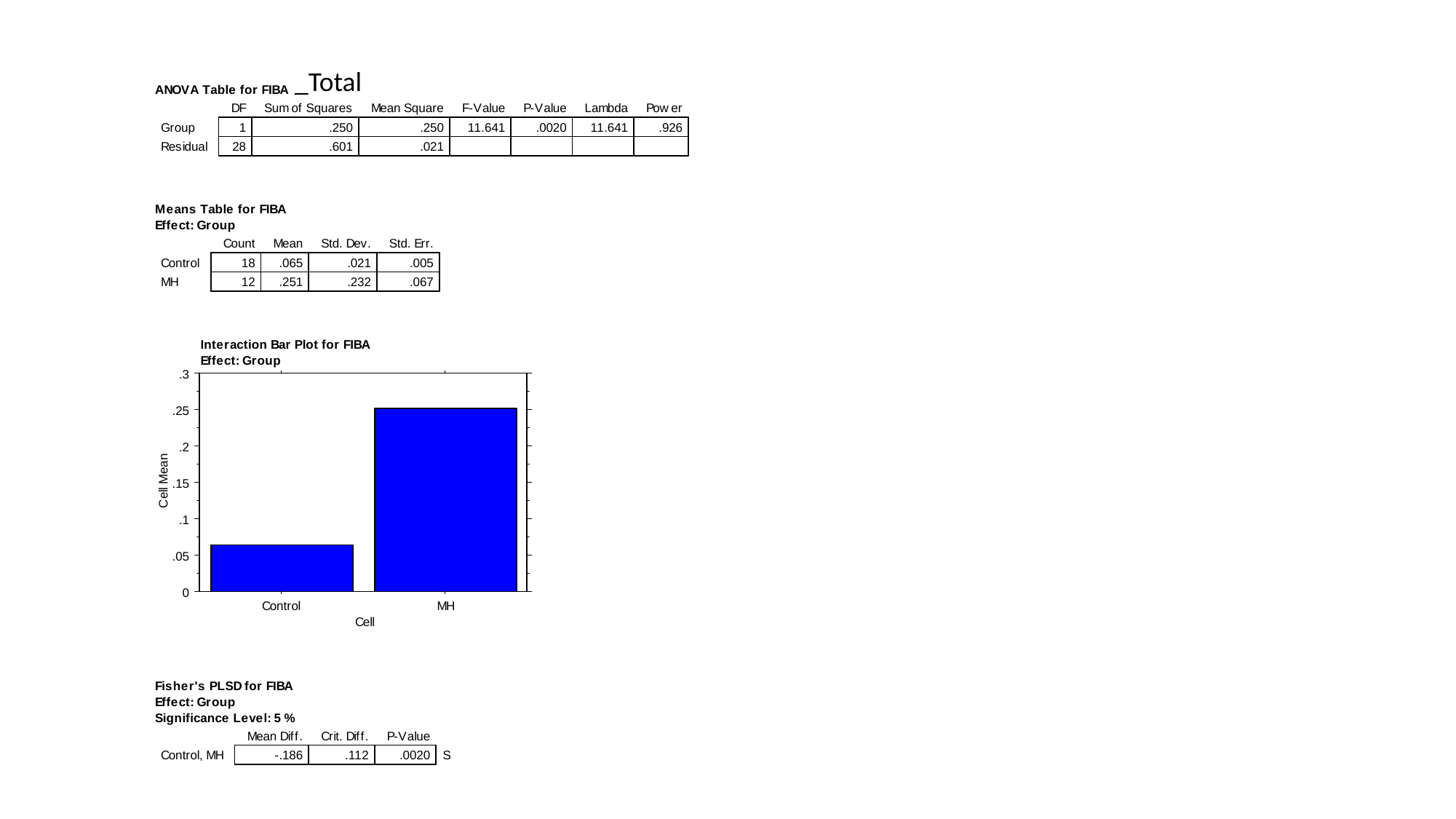

_Total

## Slide 8
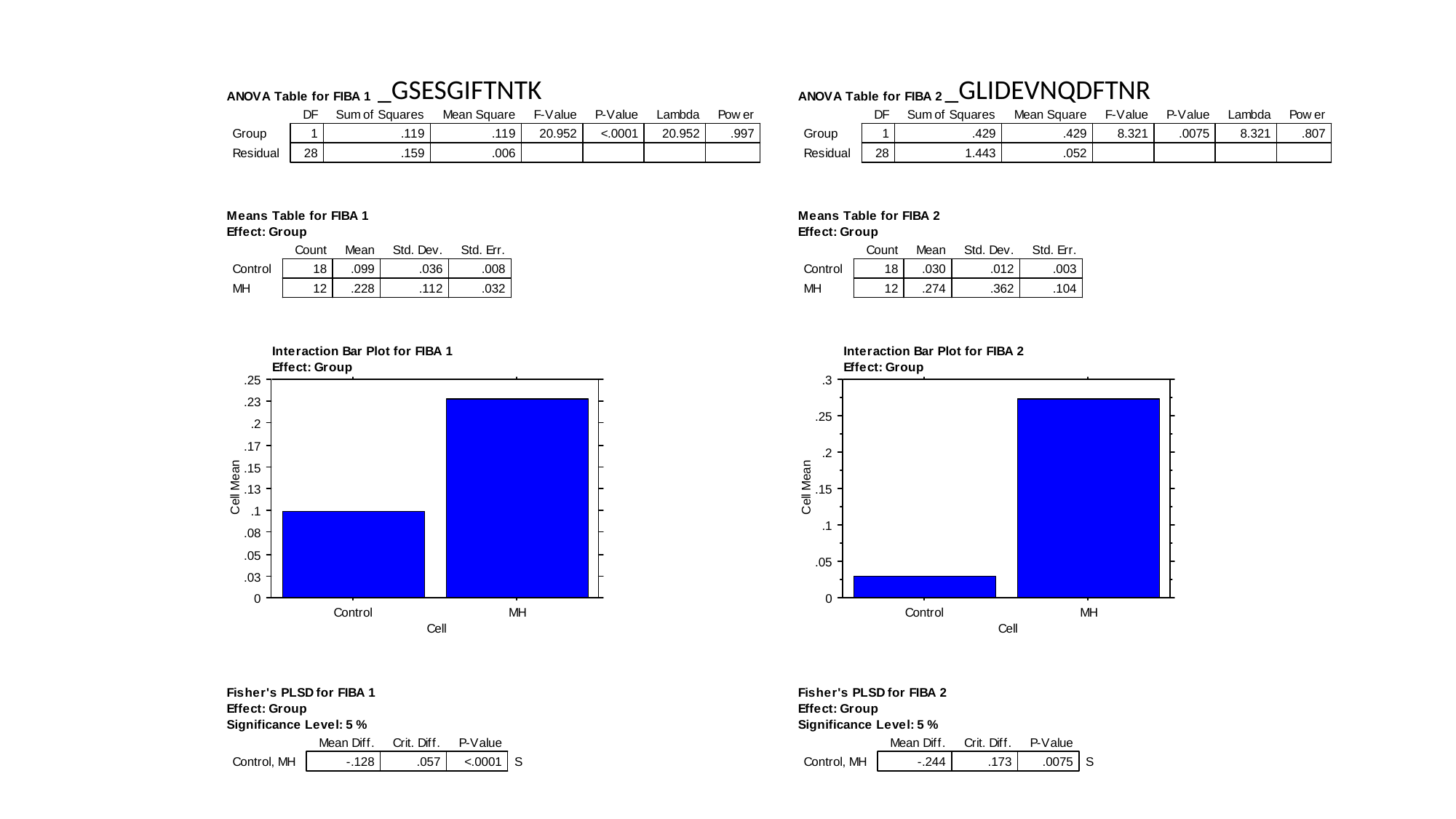

_GSESGIFTNTK
_GLIDEVNQDFTNR

## Slide 9
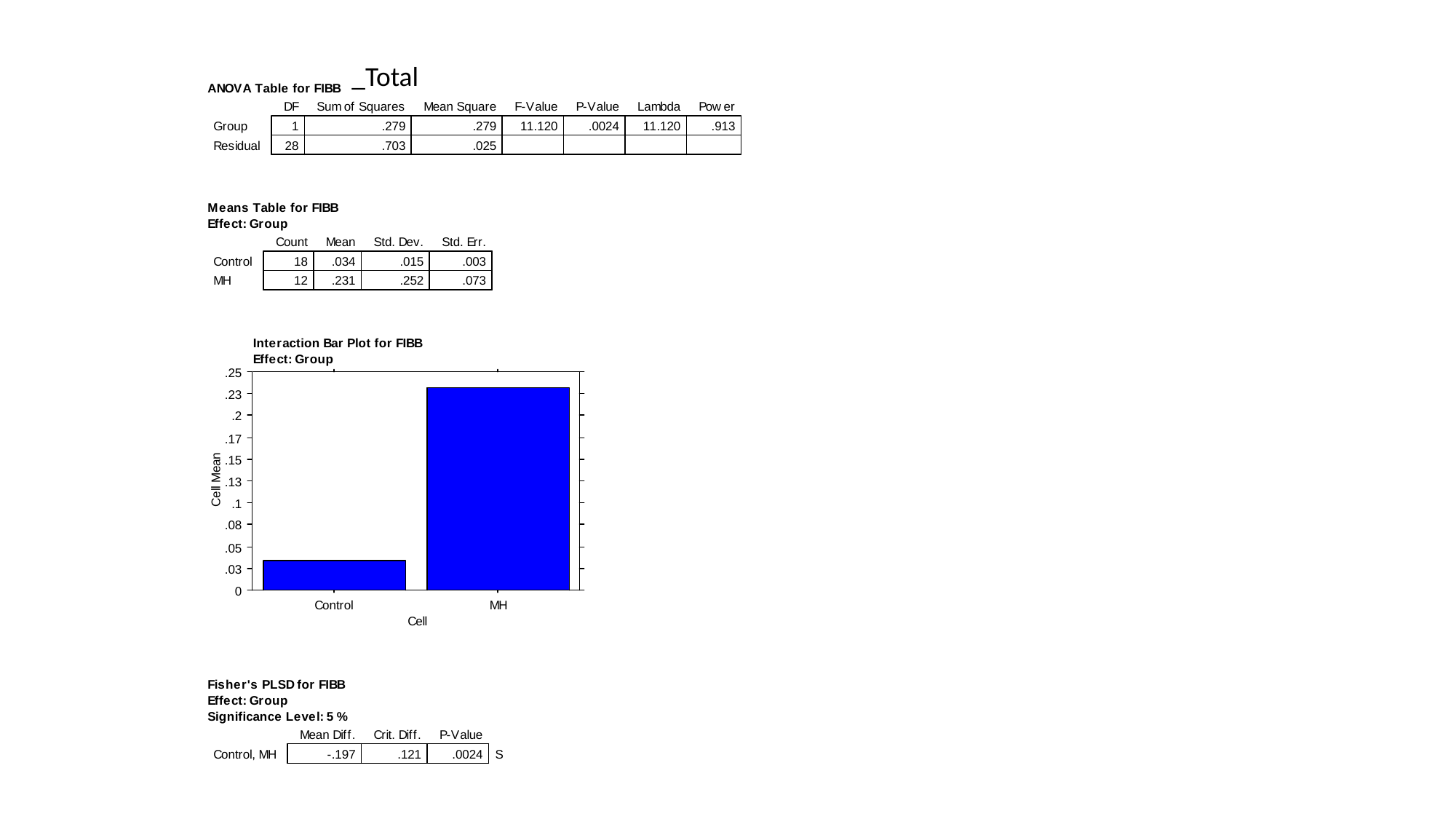

_Total

## Slide 10
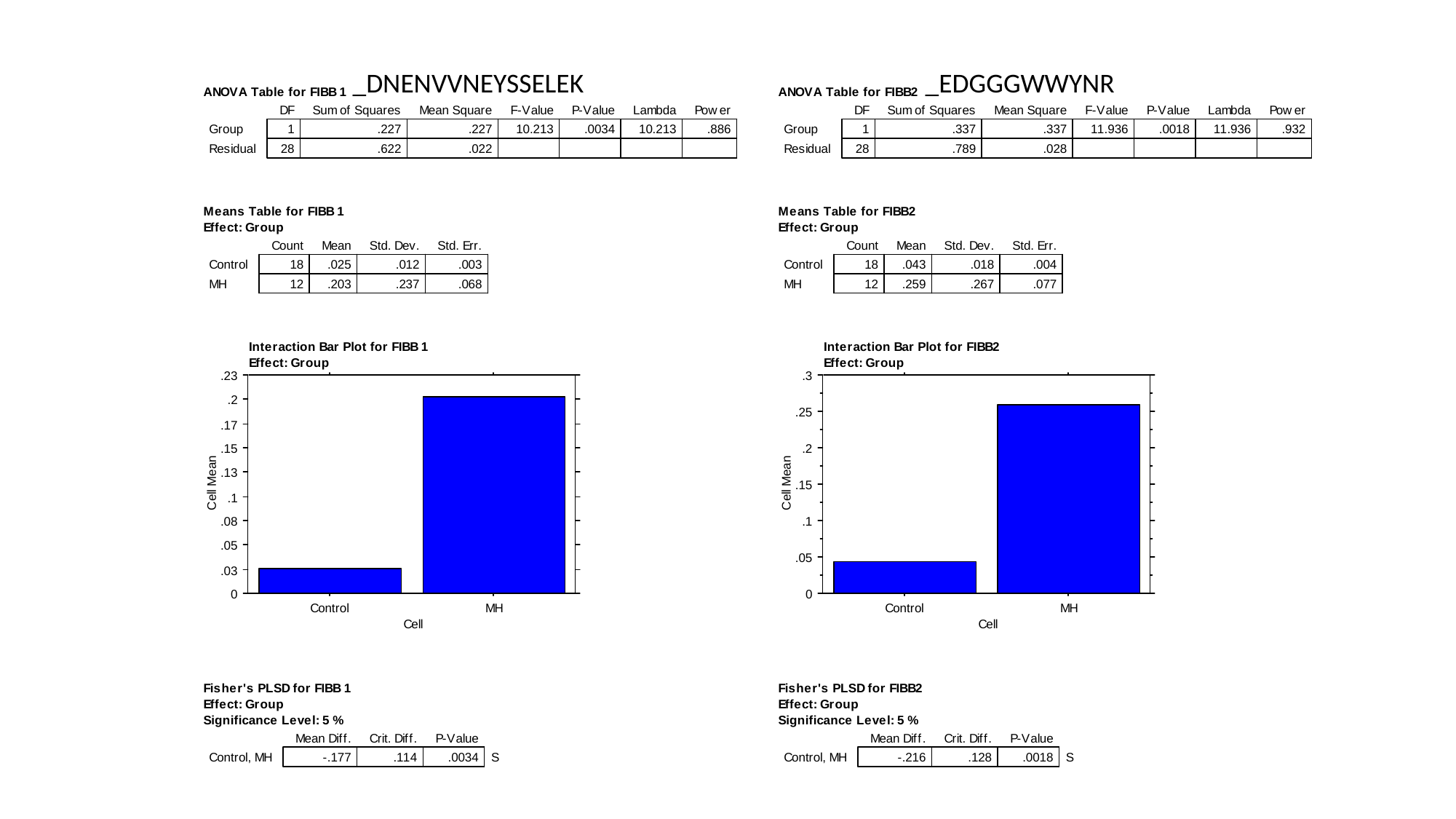

_DNENVVNEYSSELEK
_EDGGGWWYNR

## Slide 11
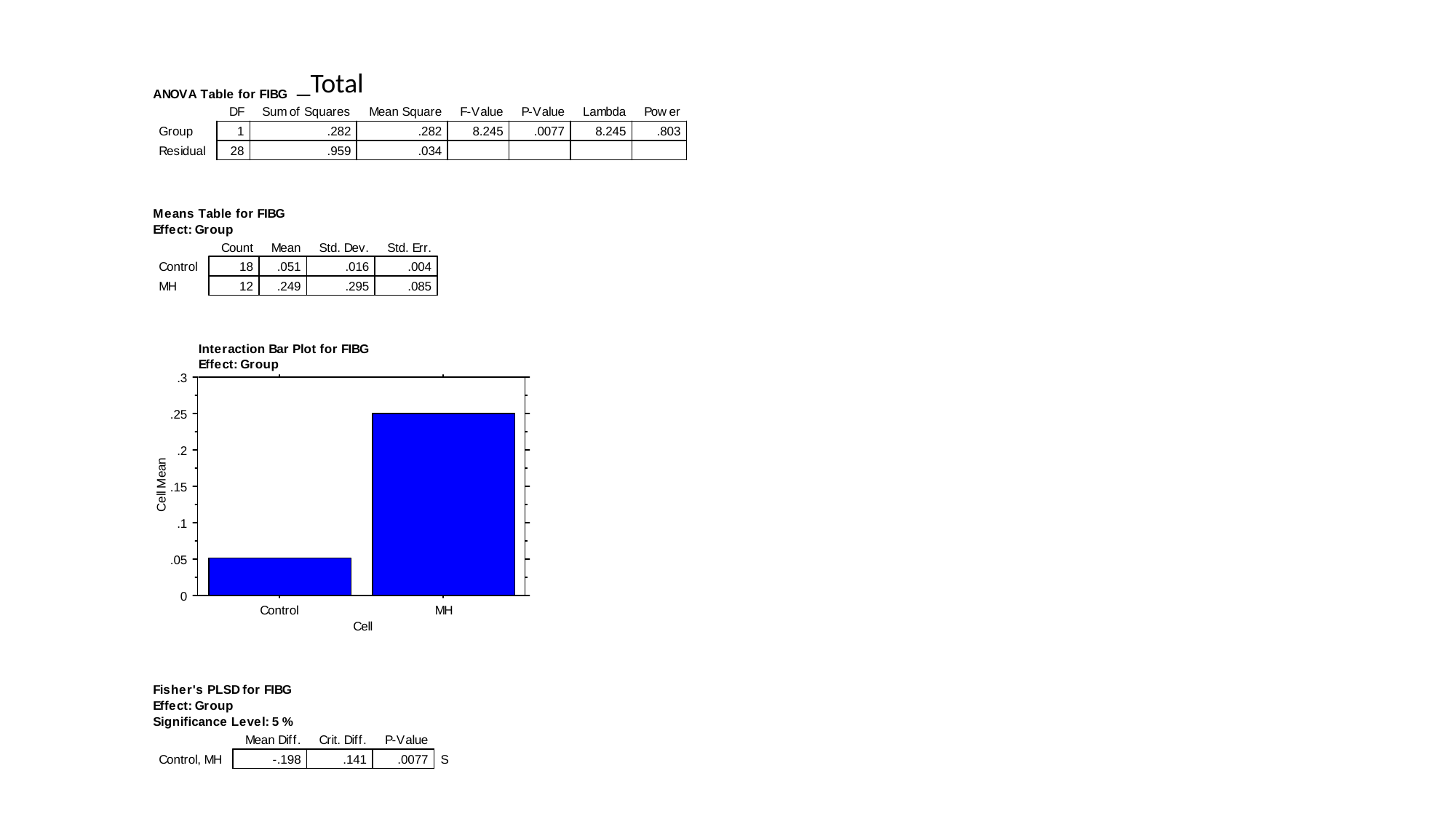

_Total

## Slide 12
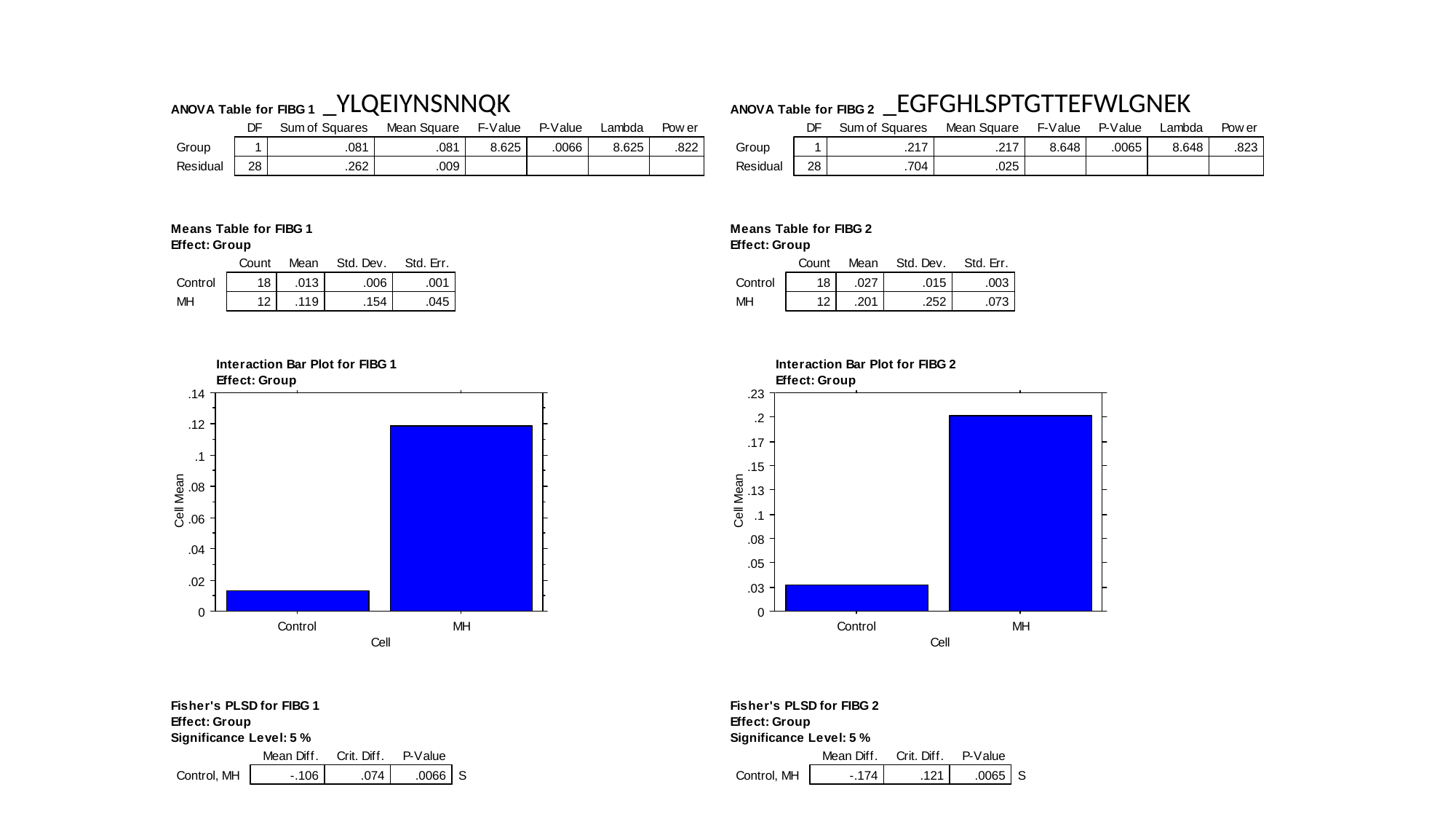

_YLQEIYNSNNQK
_EGFGHLSPTGTTEFWLGNEK

## Slide 13
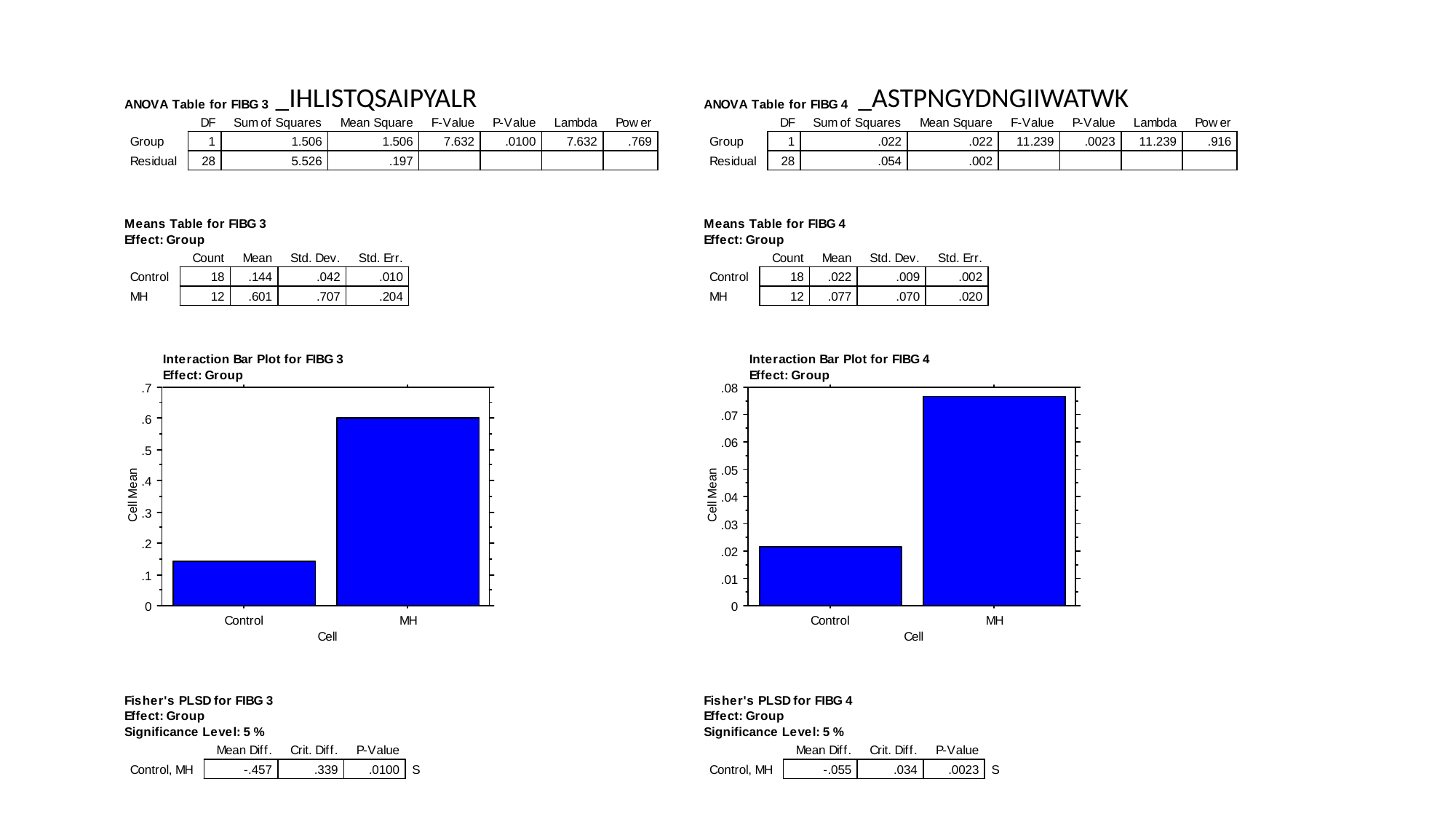

_IHLISTQSAIPYALR
_ASTPNGYDNGIIWATWK

## Slide 14
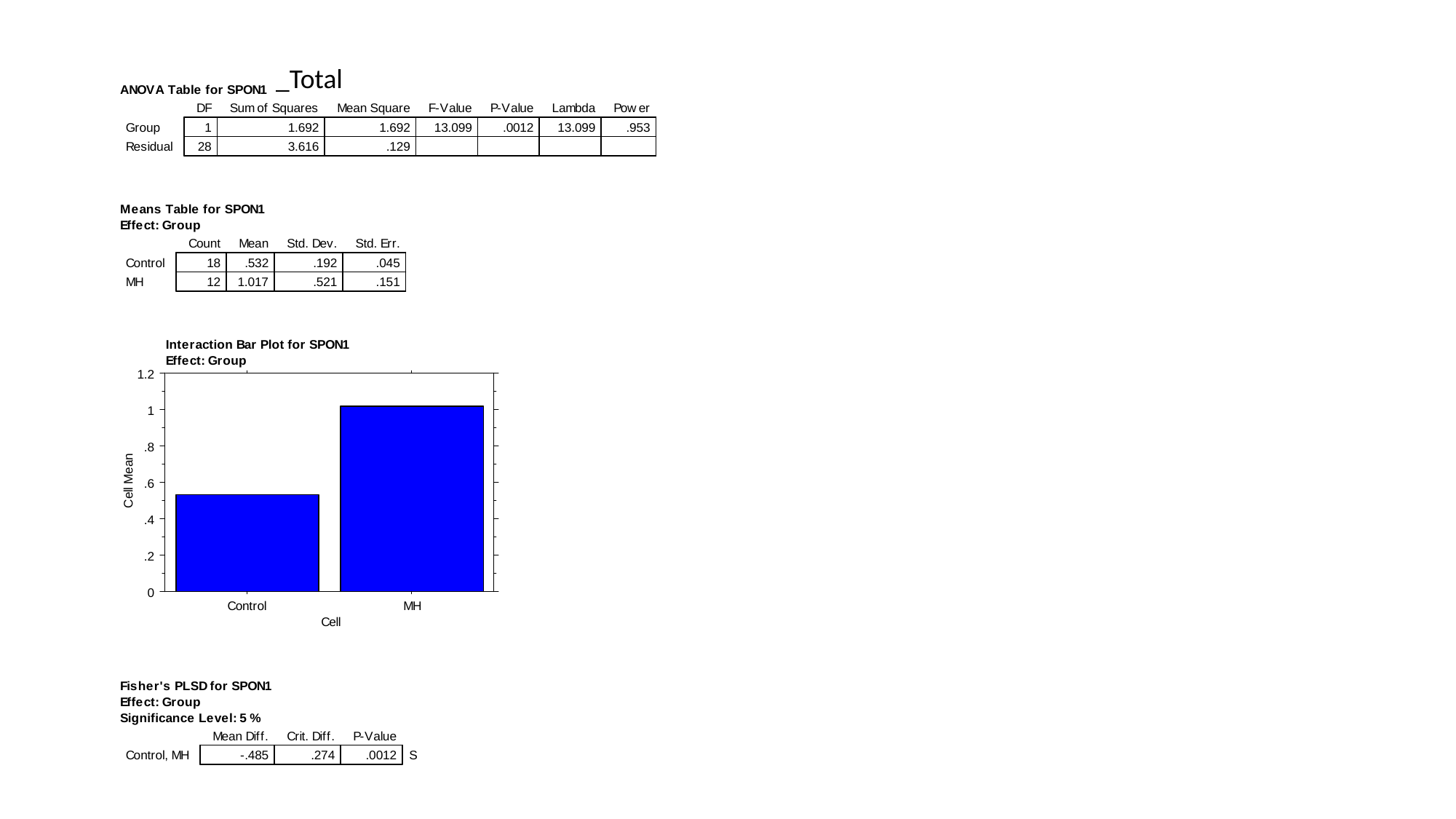

_Total

## Slide 15
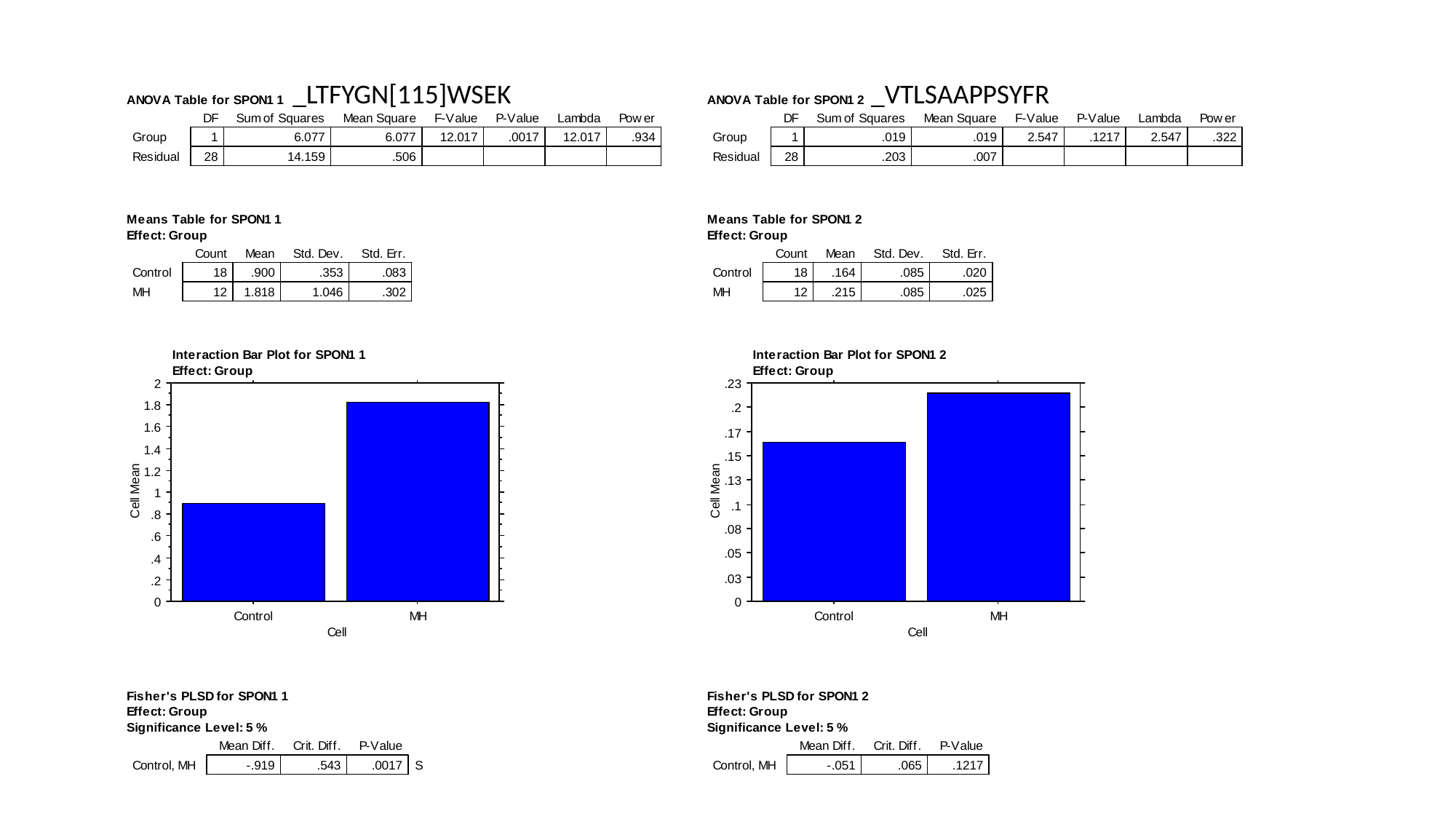

_LTFYGN[115]WSEK
_VTLSAAPPSYFR

## Slide 16
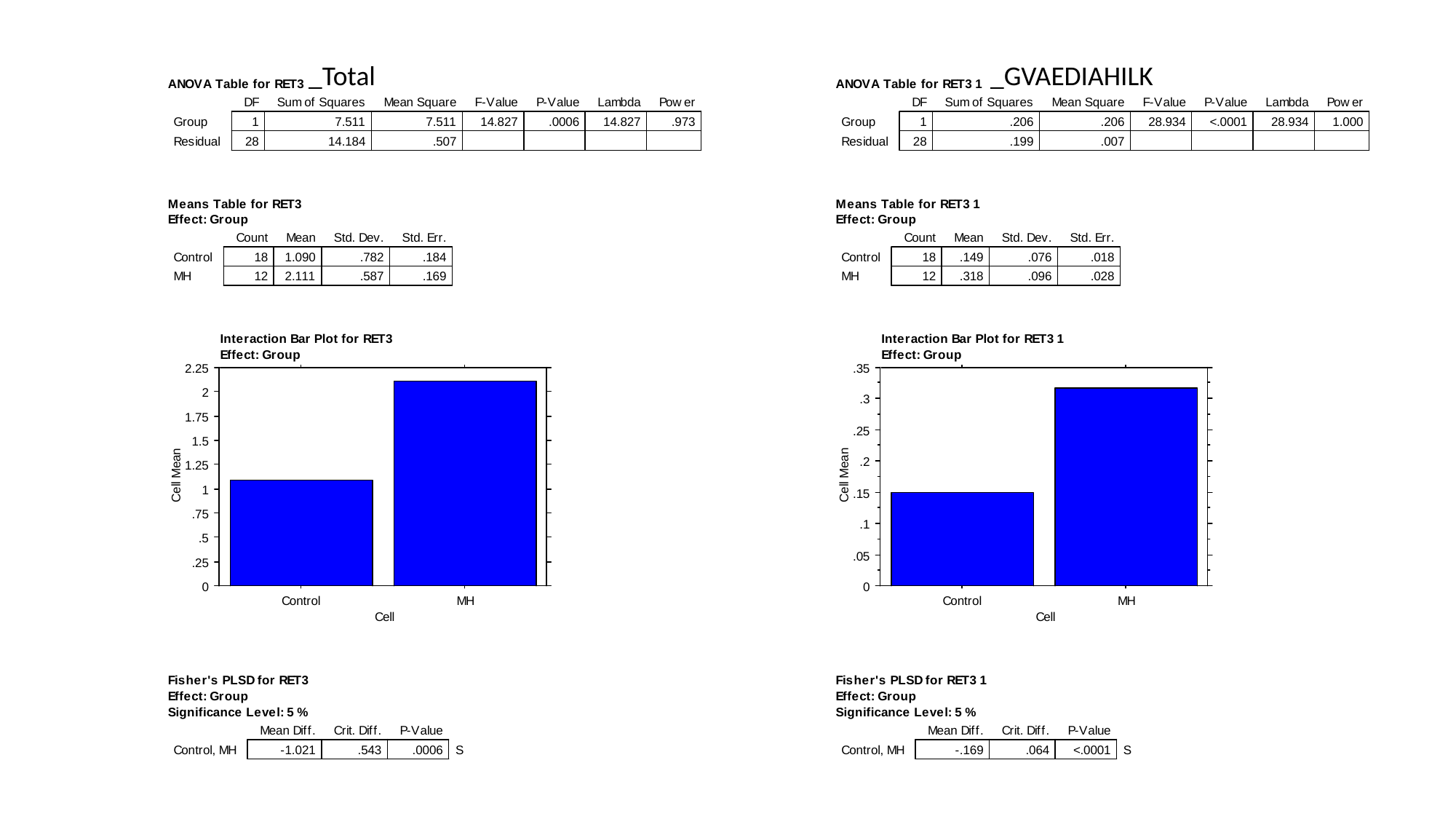

_Total
_GVAEDIAHILK

## Slide 17
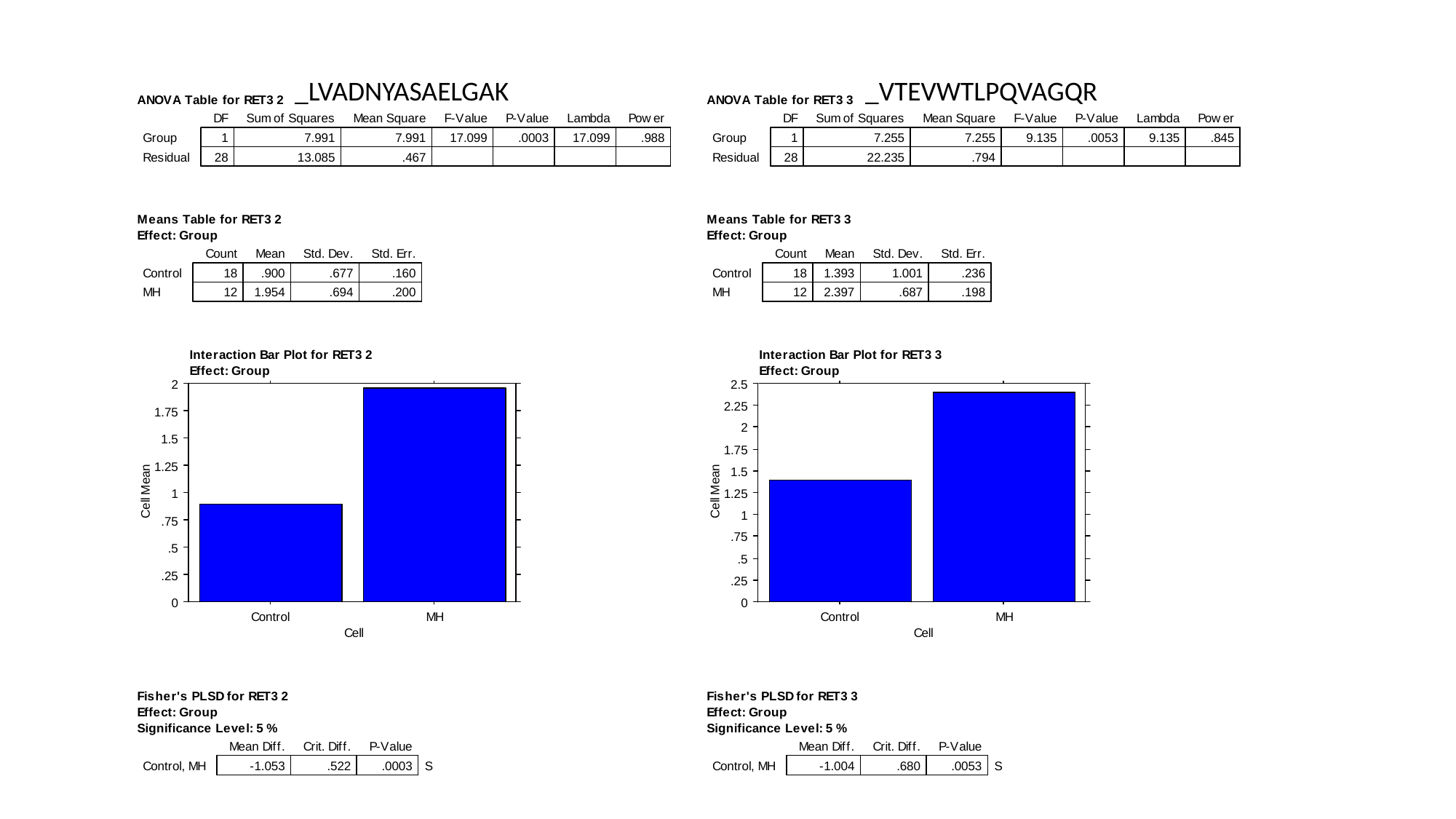

_LVADNYASAELGAK
_VTEVWTLPQVAGQR

## Slide 18
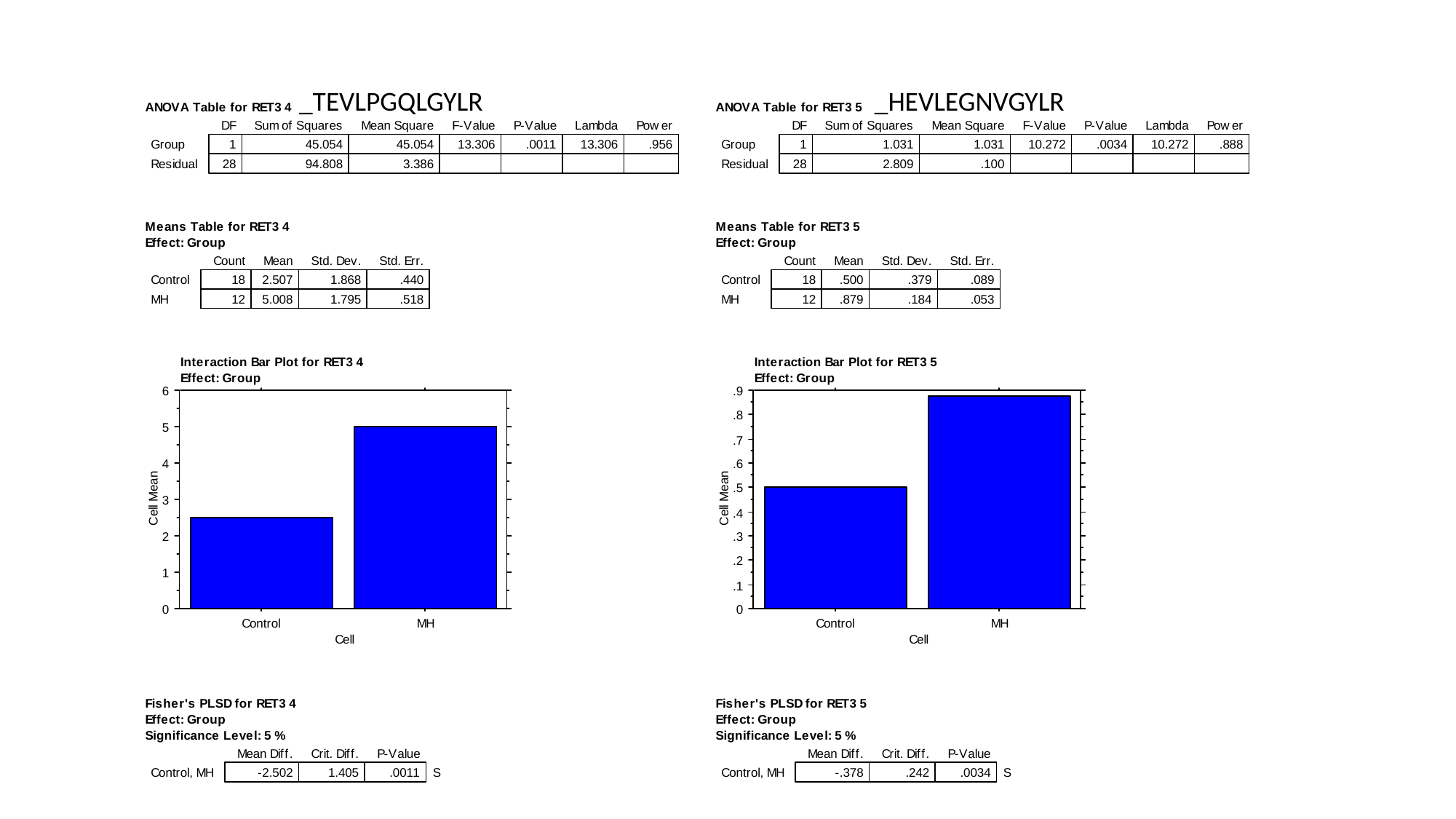

_TEVLPGQLGYLR
_HEVLEGNVGYLR

## Slide 19
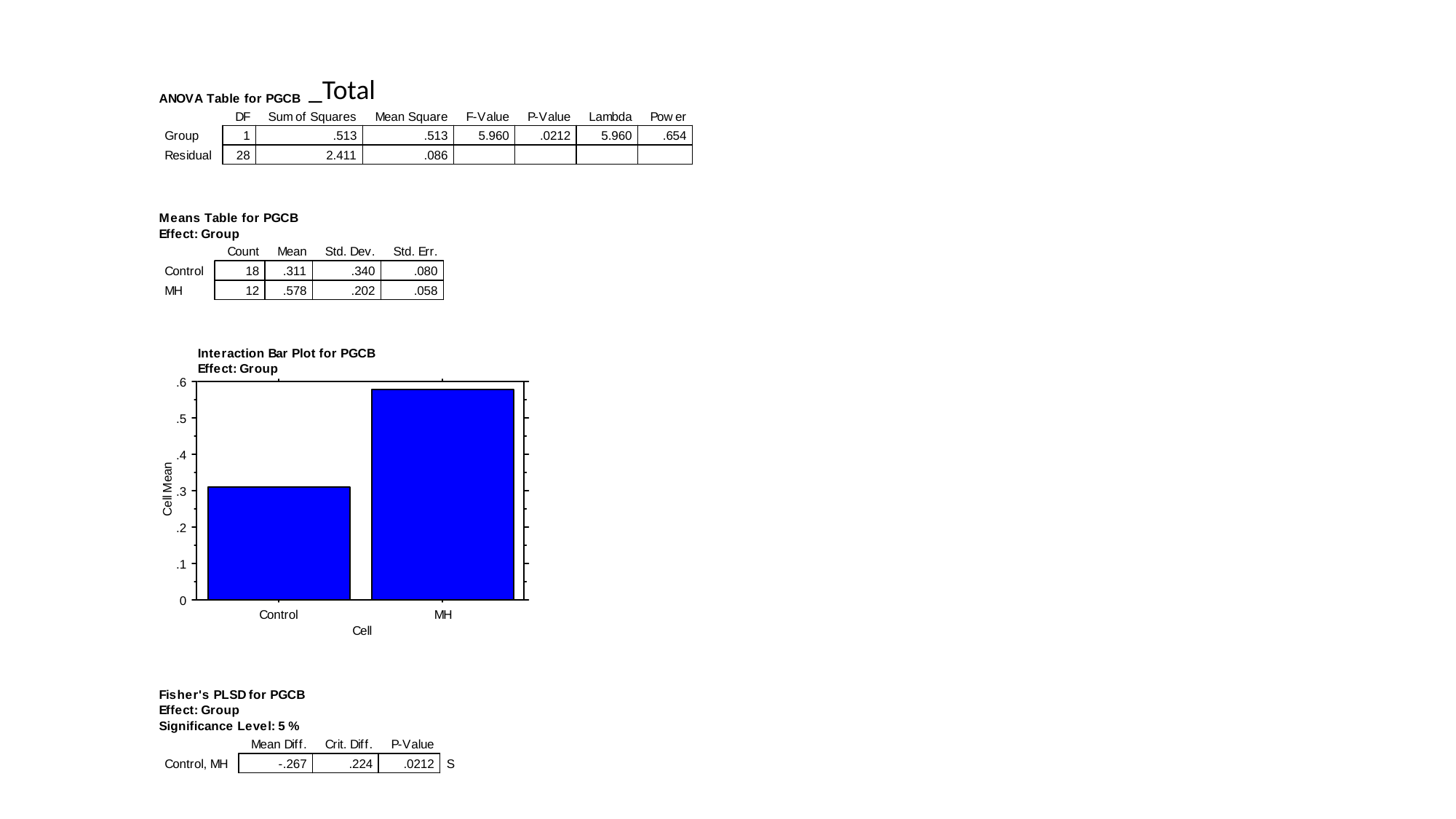

_Total

## Slide 20
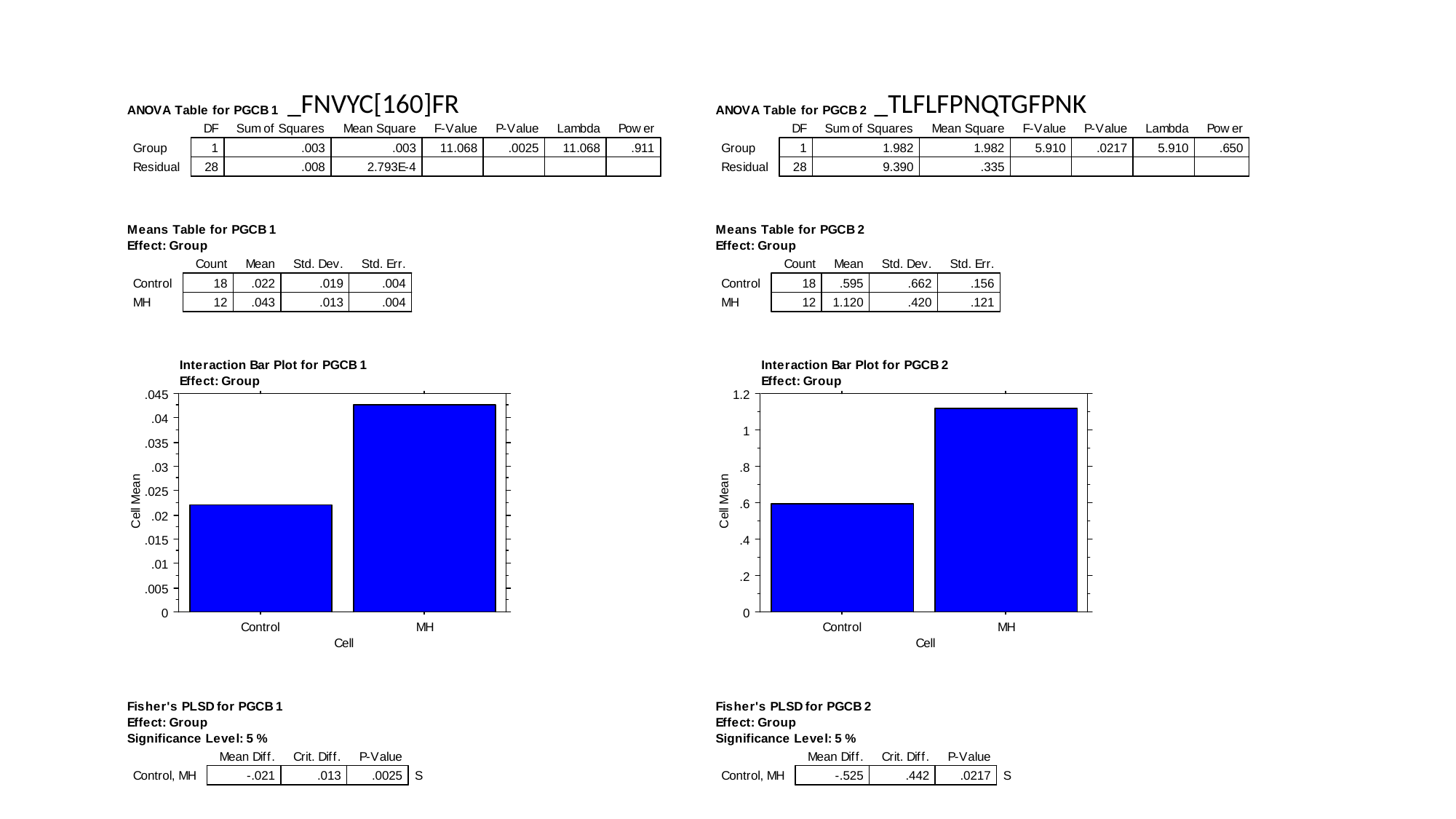

_FNVYC[160]FR
_TLFLFPNQTGFPNK

## Slide 21
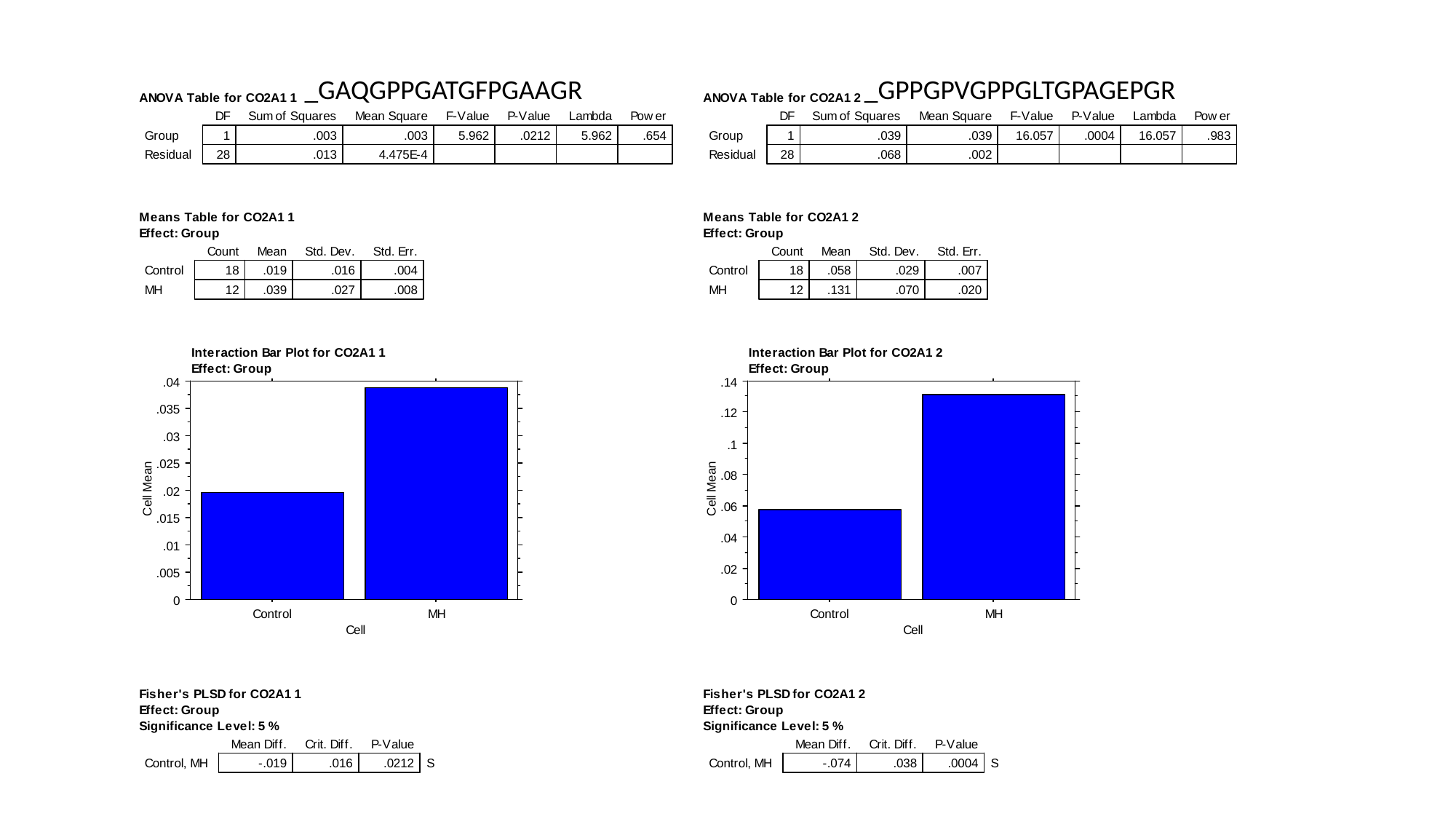

_GAQGPPGATGFPGAAGR
_GPPGPVGPPGLTGPAGEPGR

## Slide 22
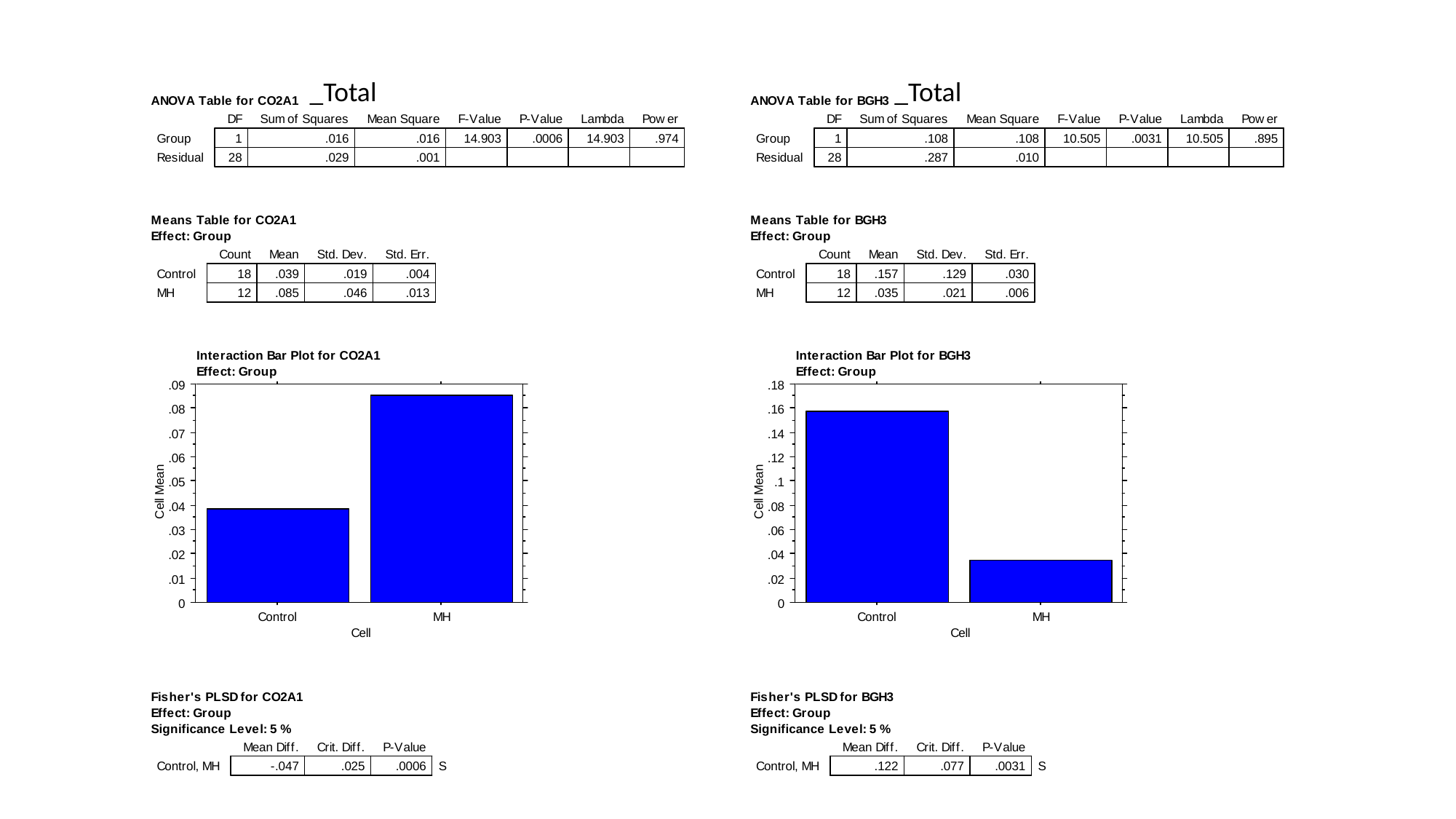

_Total
_Total

## Slide 23
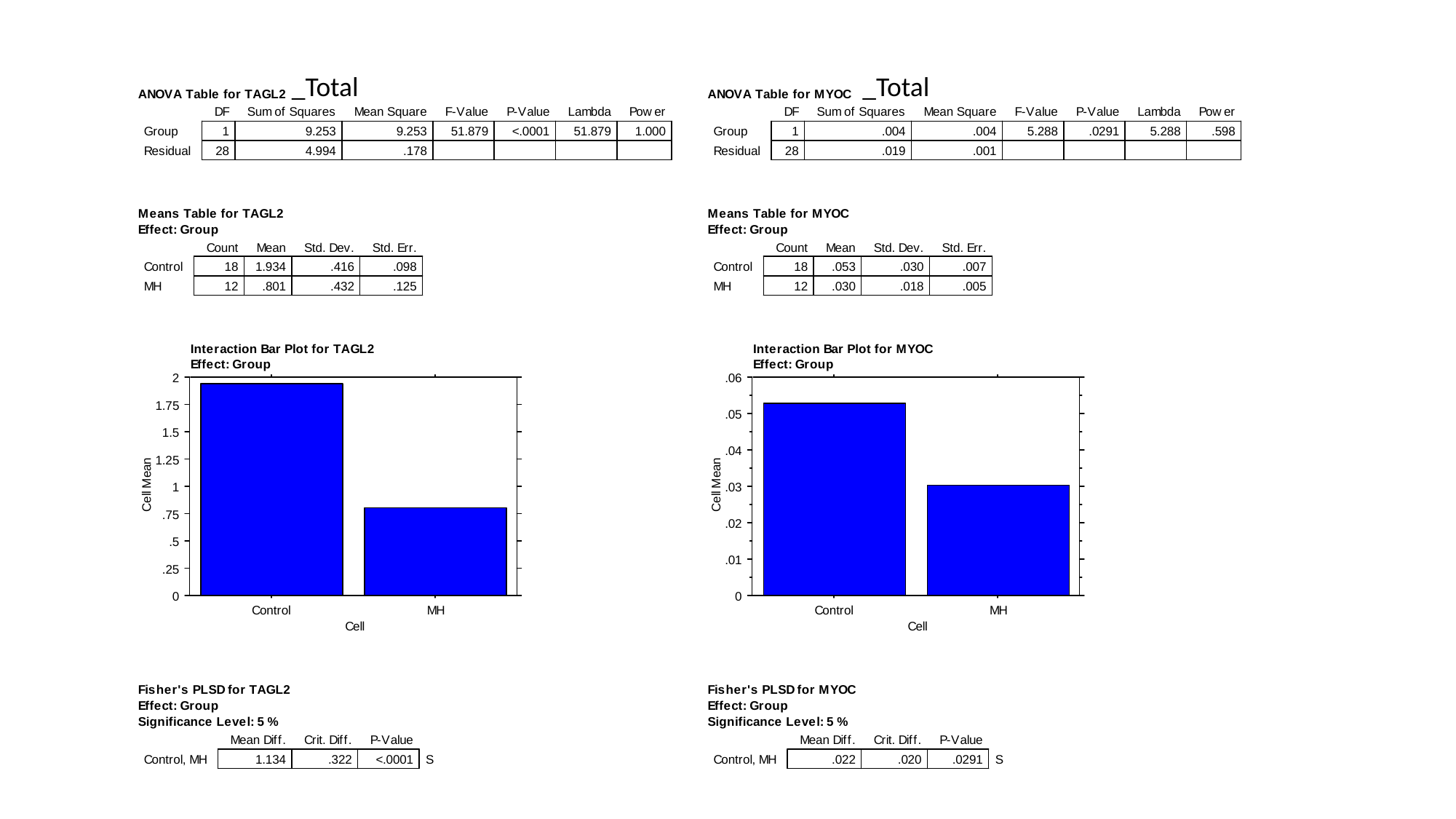

_Total
_Total

## Slide 24
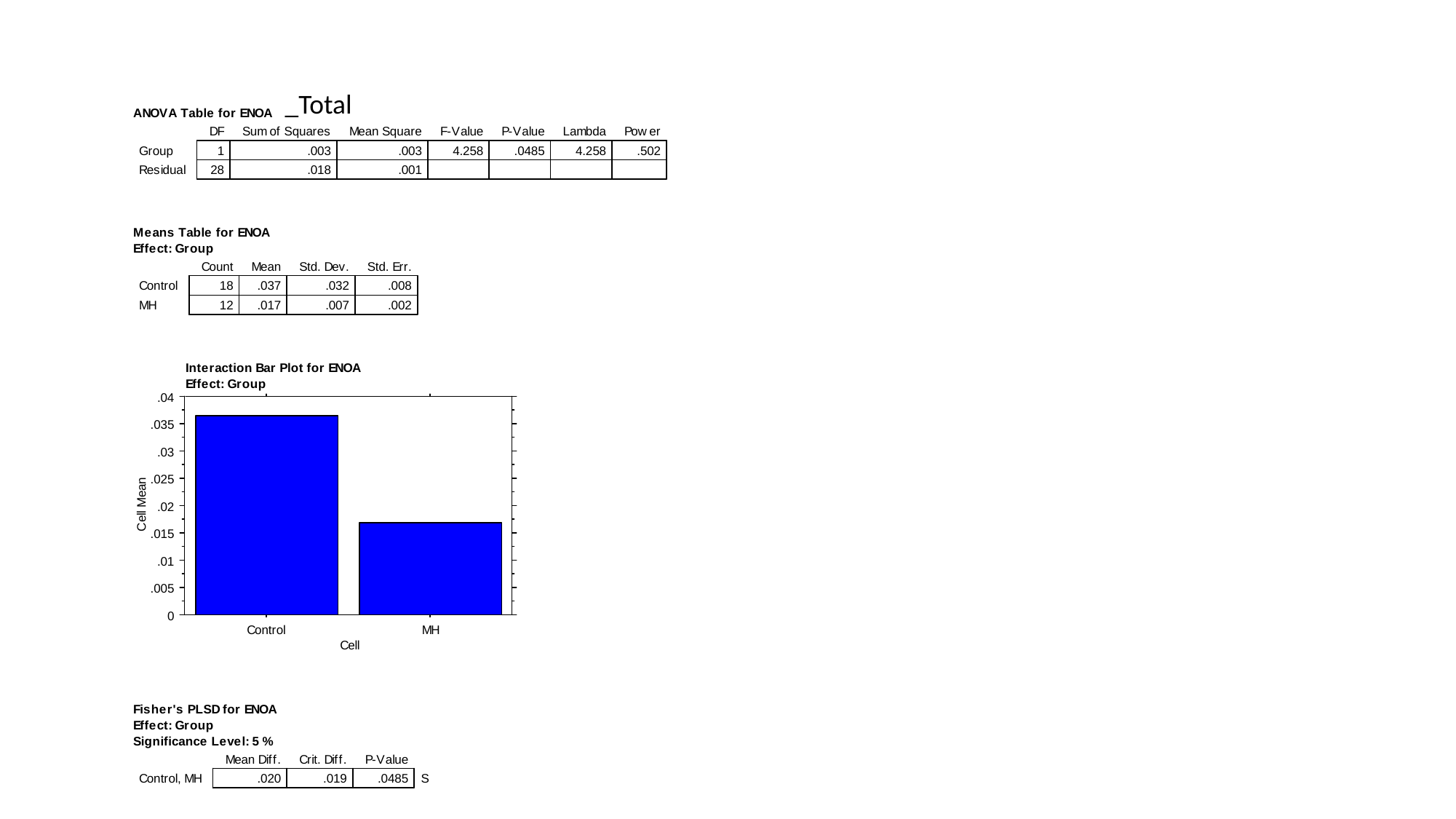

_Total

## Slide 25
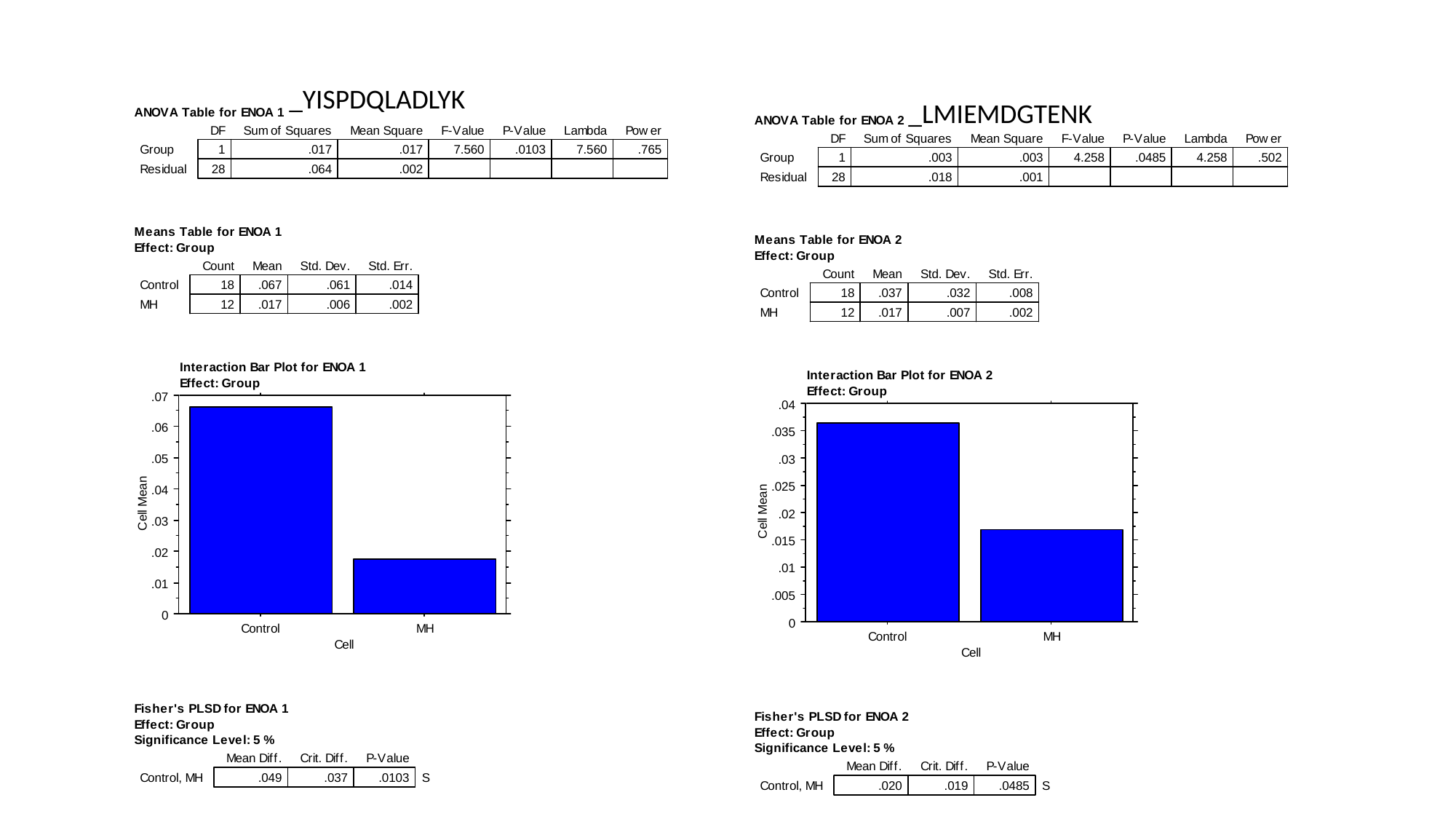

_YISPDQLADLYK
_LMIEMDGTENK
